# Metagenomic and metatranscriptomic insights into sulfate-reducing bacteria in a revegetated acidic mine wasteland

**DOI:** 10.1038/s41522-022-00333-9

**Published:** 2022-09-06

**Authors:** Jin-tian Li, Pu Jia, Xiao-juan Wang, Shu-ning Ou, Tao-tao Yang, Shi-wei Feng, Jing-li Lu, Zhou Fang, Jun Liu, Bin Liao, Wen-sheng Shu, Jie-Liang Liang

**Affiliations:** 1 Institute of Ecological Science, Guangzhou Key Laboratory of Subtropical Biodiversity and Biomonitoring, Guangdong Provincial Key Laboratory of Biotechnology for Plant Development, School of Life Sciences, South China Normal University, Guangzhou, 510631 PR China; 2 School of Life Sciences, Sun Yat-sen University, Guangzhou, 510275 PR China

**Keywords:** Soil microbiology, Metagenomics, Next-generation sequencing

## Abstract

The widespread occurrence of sulfate-reducing microorganisms (SRMs) in temporarily oxic/hypoxic aquatic environments indicates an intriguing possibility that SRMs can prevail in constantly oxic/hypoxic terrestrial sulfate-rich environments. However, little attention has been given to this possibility, leading to an incomplete understanding of microorganisms driving the terrestrial part of the global sulfur (S) cycle. In this study, genome-centric metagenomics and metatranscriptomics were employed to explore the diversity, metabolic potential, and gene expression profile of SRMs in a revegetated acidic mine wasteland under constantly oxic/hypoxic conditions. We recovered 16 medium- to high-quality metagenome-assembled genomes (MAGs) containing reductive *dsrAB*. Among them, 12 and four MAGs belonged to *Acidobacteria* and *Deltaproteobacteria*, respectively, harboring three new SRM genera. Comparative genomic analysis based on seven high-quality MAGs (completeness >90% and contamination <10%; including six acidobacterial and one deltaproteobacterial) and genomes of three additional cultured model species showed that *Acidobacteria*-related SRMs had more genes encoding glycoside hydrolases, oxygen-tolerant hydrogenases, and cytochrome c oxidases than *Deltaproteobacteria*-related SRMs. The opposite pattern was observed for genes encoding superoxide reductases and thioredoxin peroxidases. Using VirSorter, viral genome sequences were found in five of the 16 MAGs and in all three cultured model species. These prophages encoded enzymes involved in glycoside hydrolysis and antioxidation in their hosts. Moreover, metatranscriptomic analysis revealed that 15 of the 16 SRMs reported here were active in situ. An acidobacterial MAG containing a prophage dominated the SRM transcripts, expressing a large number of genes involved in its response to oxidative stress and competition for organic matter.

## Introduction

Sulfate-reducing microorganisms (SRMs) are characterized by their ability to grow with energy derived from the reduction of sulfate to sulfide^1,2^. The canonical dissimilatory sulfate reduction pathway in SRMs is driven by a set of enzymes^3^, including sulfate adenylyltransferase (Sat), adenylyl sulfate reductase (AprBA), and dissimilatory sulfite reductase (DsrAB). More specifically, Sat and AprBA, encoded by *sat* and *aprBA*, cooperate to complete the reduction of sulfate to sulfite^2^. Reductive DsrAB, encoded by reductive *dsrAB*, interacts with DsrC to reduce sulfite to sulfide^4,5^, a rate-limiting step in the biogeochemical cycle of S on Earth^6,7^.

Due to the difficulty in isolating pure cultures of SRMs^8^, the functional genes *aprBA* and reductive *dsrAB* have been widely employed to explore SRM diversity in the environment^9–13^. A striking example is the work of Vigneron et al.^13^. The authors found 167,397 different species-level *dsrB* OTUs affiliated with 47 different families in 14 different ecosystems. Among these OTUs, ~99% were previously not detected, greatly improving our knowledge of the global species-level biodiversity of SRMs.

Recently, a new trend in SRM research has been the application of genome-centric metagenomics^14–18^. One of the most important advantages of this approach lies in the recovery of near-complete genomes representing species-level microorganisms of interest^19^. This advantage not only allows the identification of previously uncultured microorganisms of interest but also provides insights into the metabolic potentials of microorganisms of interest^20–22^, although some findings from metagenomics studies cannot be definitely confirmed until pure cultures of microorganisms of interest are obtained. Nonetheless, recent studies on MAGs containing reductive *dsrAB* from environments have revealed that: (1) eight prokaryotic phyla that were not previously reported to have SRMs harbor the canonical functional genes required for dissimilatory sulfate reduction^14^; (2) two acidobacterial MAGs encoding reductive DsrAB but not Sat and AprBA contain sulfite-producing enzyme genes that allow them to use organosulfonates as growth substrates^15^; and (3) viruses can infect SRMs in wetland sediments and thus likely affect the functions of their hosts in previously unknown ways^16^. Furthermore, although very rare, genome-centric metatranscriptomic evidence suggests that previously unknown *Acidobacteria-*related SRMs play an important role in sulfate reduction in anoxic peat soils^15^.

However, little is known about SRMs in terrestrial environments^2^. This situation represents an incomplete understanding of microorganisms responsible for the terrestrial part of the global S cycle, as the geographic distribution of sulfate-rich soils is not restricted to aquatic environments^23^. Many natural processes (e.g., prolonged droughts) and anthropogenic interventions (including mining operations) can lead to the distribution of sulfate-rich soils in terrestrial environments^23^. A major distinction between terrestrial and aquatic sulfate-rich soils lies in the constantly oxic/hypoxic conditions associated with the former, although oxic/hypoxic conditions can temporarily exist in the latter^24^. Such discrepancies, however, should not preclude the occurrence and activity of SRMs in terrestrial sulfate-rich soils.

The functioning of SRMs in temporarily oxic/hypoxic aquatic environments has long been recognized^25,26^. Several cultivated model SRMs in *Deltaproteobacteria* are known to possess a variety of enzymes enabling them to tolerate oxidative stress^24^. A recent study obtained the pure SRM cultures (i.e., *Desulfovibrio vulgaris* strains) that could grow using energy derived from oxygen reduction^27^. In contrast, the existing literature contains only a few lines of evidence demonstrating the occurrence or activity of SRMs in terrestrial sulfate-rich soils under constantly oxic/hypoxic conditions^28^. A previous study showed that a majority of potential SRMs in oxic/hypoxic mine tailings (i.e., the materials left after extraction and beneficiation of ores) were affiliated with *Firmicutes* and *Deltaproteobacteria*^29^. In addition, two acidobacterial MAGs retrieved from an acidic mine site were found to encode canonical enzymes required for sulfate reduction^14^. Nonetheless, no information on the transcriptomes of SRMs under constantly oxic/hypoxic conditions has been reported^14^.

Given the abovementioned information, we hypothesized that diverse SRMs can be alive in sulfate-rich mine wastelands under constantly oxic/hypoxic conditions but their survival strategies likely differ considerably between lineages. To test our hypothesis, we employed genome-centric metagenomics and metatranscriptomics to characterize SRMs in a revegetated acidic mine wasteland. Before revegetation, the wasteland (a mine tailings pond; pH ~2.5) was abandoned and drained for eight years. A promising remediation technology termed ‘phytostabilization’ was used to revegetate the wasteland^30^, thereby facilitating in situ stabilization of tailings and metal contaminants. Approximately 4000 m^2^ of vegetation was established on the wasteland, and it increasingly flourished as time progressed^30^. This revegetated mine wasteland was selected because: (1) it was a representative case illustrating the effectiveness of phytostabilization for remediating sulfate-rich mine wastelands distributed globally; and (2) it consisted of different habitats with soil oxidation-reduction potential (Eh) values varying from ~180–680 mV^31^, a representative Eh range encountered in terrestrial environments under constantly oxic/hypoxic conditions^32^.

## Results

### MAGs harboring reductive *dsrAB*

**Fig. 1:**
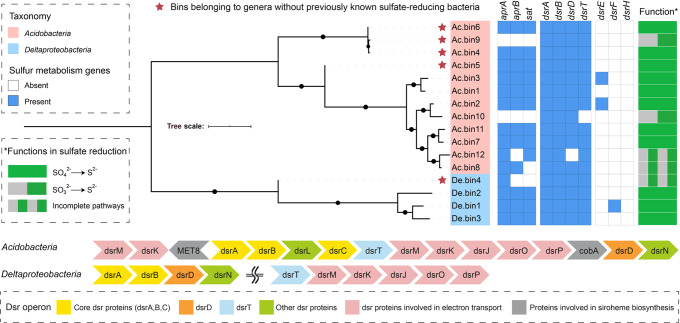
Analysis of dissimilatory sulfate reduction genes in metagenome-assembled genomes (MAGs) from revegetated acidic mine wastelands. Sixteen medium- to high-quality MAGs (completeness >50% and contamination <10%) were included in the analysis. The functions of the MAGs in sulfate reduction were predicted according to the presence and/or absence of key genes for the pathway. A representative organization of dissimilatory sulfate reduction genes on the MAGs belonging to *Acidobacteria* and *Deltaproteobacteria* is displayed, respectively. Tree scale bar = 1. Additional details are provided in Supplementary Tables 1 and 2.

We identified 50 DsrAB protein sequences in 982 medium- to high-quality MAGs (completeness >50% and contamination <10%) from 18 metagenomes from the revegetated acid mine wasteland, which were published elsewhere^31^. Sixteen of these sequences belonged to the reductive bacterial-type DsrAB family (Supplementary Fig. 1). Accordingly, 16 reductive *dsrAB*-containing MAGs were retrieved, with 12 from *Acidobacteria* and four from *Deltaproteobacteria* (Fig. 1 and Supplementary Table 1).

The 12 acidobacterial MAGs were all affiliated with subdivision 1 of *Acidobacteria* (Supplementary Fig. 2). Among them, three (i.e., Ac.bin4, Ac.bin9, and Ac.bin6) formed a monophyletic clade and had an average amino acid identity (AAI) of 63% with its closest relative (*Granulicella tundricola* MP5ACTX9)^33^. Similarly, Ac.bin5 formed a monophyletic clade and had AAIs of 56–62% with its closest relatives. The four acidobacterial MAGs mentioned above likely represented two new SRM genera, given that no SRMs from *Acidobacteria* have been successfully cultivated and that the currently known acidobacterial MAGs containing *dsrAB*^15^ were not affiliated with the genera represented by the four MAGs (Supplementary Fig. 2).

Three out of the four reductive *dsrAB*-containing deltaproteobacterial MAGs were affiliated with the well-known SRM genus *Desulfovibrio* (Supplementary Table 1). However, the remaining one (i.e., De.bin4) from the family *Syntrophobacteraceae* formed a monophyletic clade and could not be assigned to a specific genus with references available (Supplementary Fig. 3). Specifically, De.bin4 had AAIs of 54–60% with its closest relatives. Therefore, we inferred that it belonged to a new genus.

The Dsr operon structures of *Acidobacteria* were different from those of *Deltaproteobacteria* (Fig. 1). Multiple alignments of the DsrD and DsrT sequences with published references confirmed the highly conserved residues (Supplementary Figs. 4 and 5), indicating that these proteins were likely functional. According to the common patterns for determining the direction of dissimilatory S metabolism for uncultivated microorganisms^14^, 11 MAGs (eight from *Acidobacteria* and three from *Deltaproteobacteria*) in this study encoded a complete pathway for the reduction of sulfate to sulfide (Fig. 1 and Supplementary Table 2). Notably, seven high-quality MAGs (completeness >90% and contamination <10%) of SRMs (six acidobacterial and one deltaproteobacterial, Supplementary Table 1) were obtained and thus further analyzed for their metabolism. For comparison, the complete genomes of one aerobic acidobacterial species (i.e., *Terracidiphilus gabretensis* S55, non-SRM)^34^, *Desulfovibrio vulgaris* Hildenborough (an oxygen-tolerant cultured model SRM), and *Desulfococcus multivorans* DSM 2059 (an oxygen-sensitive cultured model SRM)^35^ were also chosen for functional analysis.

### Glycoside hydrolysis of SRMs

**Fig. 2:**
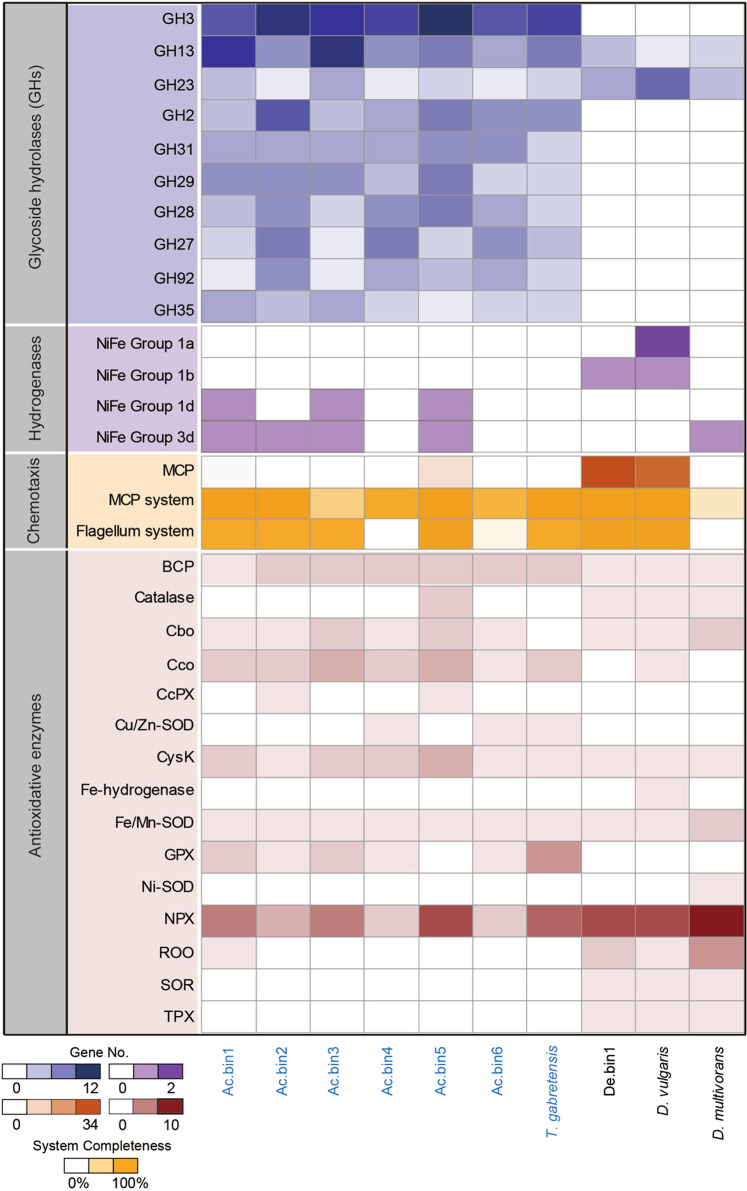
Comparative analysis of selected metabolic potentials of sulfate-reducing microorganisms (SRMs). Seven high-quality MAGs (completeness >90% and contamination <10%) of SRMs were included in the analysis. For comparison, the complete genomes of one aerobic acidobacterial species (i.e., *Terracidiphilus gabretensis* S55, non-SRM), *Desulfovibrio vulgaris* Hildenborough (an oxygen-tolerant cultured model SRM), and *Desulfococcus multivorans* DSM 2059 (an oxygen-sensitive cultured model SRM) were chosen. The names of acidobacterial genomes are in blue. The 10 most abundant glycoside hydrolase families across the genomes are shown. Those hydrogenase subgroups that are known to be involved in sulfate reduction or to be oxygen tolerant are listed. MCP methyl-accepting chemotaxis protein, BCP bacterioferritin comigratory protein, Cbo cytochrome bd oxygen reductase, Cco cytochrome c oxidase, CcPx cytochrome c peroxidase, SOD superoxide dismutase, CysK cysteine synthase, GPX glutathione peroxidase, NPX NADH peroxidase, ROO rubredoxin-oxygen oxidoreductase, SOR superoxide reductase, TPX thioredoxin peroxidase. Additional details are presented in Supplementary Tables 3–8.

Seventy-one glycoside hydrolase (GH) families were encoded by the 10 focal genomes (including six high-quality acidobacterial MAGs and one high-quality deltaproteobacterial MAG, as well as the complete genomes of one cultured acidobacterial species and two cultured deltaproteobacterial SRMs; Supplementary Table 3). Among them, all but one family (i.e., GH50) was not found in the seven acidobacterial genomes. In contrast, the three deltaproteobacterial genomes encoded only 10 GH families. Judging from the average number of genes encoding a given GH family per genome, GH3, GH13, GH23, GH2, GH31, GH29, GH28, GH27, GH92, and GH35 were the 10 most abundant genes across all 10 focal genomes (Fig. 2). Except for GH23, these abundant GH families were largely represented by acidobacterial genomes. A striking example was GH3, which was encoded by 9–13 genes in each acidobacterial genome but by only one gene per deltaproteobacterial genome (Fig. 2).

### Hydrogen metabolism of SRMs

Genes encoding eight groups of hydrogenases (including groups A1 and A2 of [FeFe]-hydrogenase and groups 1a, 1b, 1d, 3d, 4c, and 4e of [NiFe]-hydrogenase) were identified on the 10 focal genomes (Supplementary Table 4). Among them, [FeFe]-hydrogenase was encoded only by *D. vulgaris*. These results were consistent with those of Hausmann et al.^15^, who showed that MAGs of *Acidobacteria*-related SRMs harbored genes encoding groups 1 (excluding 1 h), 3, and 4 of [NiFe]-hydrogenase. Examination of individual genomes showed that they differed considerably in the total number of hydrogenase genes (Supplementary Table 4). The genome of *D. vulgaris* contained up to seven hydrogenase genes, while three acidobacterial genomes (i.e., Ac.bin6, Ac.bin4 and *T. gabretensis*) lacked such genes. Despite this, seven out of the eight genes encoding oxygen-tolerant hydrogenases (i.e., groups 1d and 3d of [NiFe]-hydrogenase^36^) were identified in the remaining four acidobacterial genomes (Fig. 2).

### Respiratory chain of SRMs

All 10 selected genomes encoded the major components of the respiratory chain (Supplementary Table 5). Specifically, the (near) complete operons for NADH dehydrogenase 1 (lacking in *D. vulgaris* and *D. multivorans*), NADH dehydrogenase 2 (lacking in Ac.bin6 and Ac.bin4), succinate dehydrogenase, quinol–cytochrome-c reductase, high-affinity terminal oxidase (lacking in *T. gabretensis*), low-affinity terminal oxidase (lacking in *D. multivorans*), and F-type ATP synthase were detected. Remarkably, *bd*-type terminal oxidase was encoded by all the focal genomes except that of *T. gabretensis*, while *cbb3*-type terminal oxidase was detected in only an acidobacterial MAG (i.e., Ac.bin3, Supplementary Table 5).

### Methyl-accepting chemotaxis protein system of SRMs

Compared with the seven acidobacterial genomes, two *Desulfovibrio*-related genomes (i.e., De.bin1 and *D. vulgaris*) encoded many more methyl-accepting chemotaxis protein (MCPs; Fig. 2 and Supplementary Table 6), which showed a high level of sequence similarity between these two SRMs (Supplementary Fig. 6). No MCP genes were detected in Ac.bin3 and *D. multivorans*. Among the seven known MCP classes^37^, five were recorded here (including classes Ia, IIIm, IIIc, IVa, and IVb). Although the majority of the MCPs encoded by the two *Desulfovibrio*-related genomes belonged to class Ia (including clusters I and II; Supplementary Table 7), the acidobacterial MCPs were mainly from classes IVa and IVb. Remarkably, among the 10 focal genomes, Ac.bin5 was the only one that harbored both class Ia (cluster I) and class IVa MCP genes (Supplementary Table 7).

A complete set of genes encoding core chemotaxis signaling complexes (i.e., CheB, CheR, CheW, CheA, and CheY)^38^ were detected in almost all acidobacterial genomes (except Ac.bin3) and the two *Desulfovibrio-*related genomes (Fig. 2 and Supplementary Table 6). In addition, the *che* operon structure in De.bin1 was the same as that of *cheA3* in *D. vulgaris* (Supplementary Fig. 7), suggesting that it had the ability to sense sulfate as an electron acceptor and lactate as an electron donor^39^.

### Flagellar system of SRMs

A complete set of 24 core flagellar genes^40^ was identified in five acidobacterial genomes (i.e., Ac.bin1, Ac.bin2, Ac.bin3, Ac.bin5, and *T. gabretensis*) and two *Desulfovibrio*-related genomes (i.e., De.bin1 and *D. vulgaris*; Fig. 2 and Supplementary Table 6). Genes encoding highly conserved components of the type IV pilus (e.g., *pilA*) were identified in Ac.bin4 and Ac.bin6 (Supplementary Table 6), indicating that these two SRMs could move towards chemoattractants using pili-based “twitching” motility^41^. These results, together with those for the MCP system, suggested that six genomes (i.e., Ac.bin1, Ac.bin2, Ac.bin5, *T. gabretensis*, De.bin1, and *D. vulgaris*) had potential to utilize flagellum-driven chemotaxis to sense surrounding chemoattractants and relocate themselves towards favorable microenvironments.

### Antioxidative enzymes of SRMs

Among the four known enzymes involved in oxygen reduction by SRMs^24^, only cytochrome bd oxygen reductase (Cbo, EC 7.1.1.7) was encoded by all 10 focal genomes except *T. gabretensis* (Fig. 2 and Supplementary Table 8). The other three enzymes showed two contrasting patterns: (1) [Fe] hydrogenase (EC 1.12.7.2) and rubredoxin-oxygen oxireductase (ROO) were encoded largely by deltaproteobacterial genomes; and (2) cytochrome c oxidase (Cco, EC 7.1.1.9) occurred mainly in acidobacterial genomes (Fig. 2). Similarly, two opposite trends were observed for the two major enzymes responsible for eliminating superoxide anion radicals:^24^ (1) all the investigated genomes contained at least one SOD (EC 1.15.1.1) gene, although the type of SOD differed among genomes; and (2) superoxide reductase (SOR, EC 1.15.1.2) genes were present only in deltaproteobacterial genomes (Fig. 2). Note that the majority of the acidobacterial genomes lacked genes encoding catalase (EC 1.11.1.6) and thioredoxin peroxidase (Tpx, EC 1.11.1.15), while they harbored more genes encoding thioredoxin-dependent peroxiredoxin (BCP, EC 1.11.1.24), cysteine synthase (CysK, EC 2.5.1.47) and glutathione peroxidase (GPX, EC 1.11.1.9) than deltaproteobacterial genomes.

### Viruses of SRMs

**Fig. 3:**
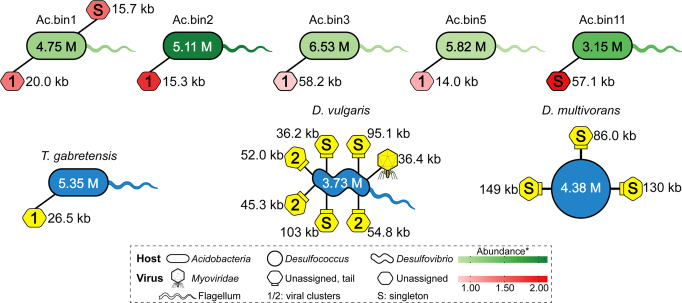
Predicted linkages between SRMs and their viruses. The 16 medium- to high-quality MAGs of SRMs were included in the analysis. For comparison, the complete genomes of *T. gabretensis*, *D. vulgaris* and *D. multivorans* were chosen. The cell shapes of the SRMs were drawn based on information from the literature and from our analysis (e.g., the presence or absence of core genes encoding the flagellum). The predicted genome sizes of SRMs and their viruses are shown. Increasing abundances of the SRMs are indicated by darker green colors. The abundance was calculated as the normalized mean coverage depth. Whenever applicable, viral shapes were drawn based on taxonomic information from the signature genes detected in the viral scaffolds. Otherwise, viruses are indicated by hexagons. The hexagons associated with a small rectangle represent tailed viruses. The number (i.e., 1 or 2) within a given virus represents a viral cluster, from which the virus was derived. That is, those hexagons filled with the same number are affiliated with the same viral cluster. Singletons represent new viruses. Increasing abundances of the acidobacterial viruses are indicated by darker red colors. Due to the lack of abundance information, the three cultured species and their viruses are shown in blue and yellow, respectively. Additional details are presented in Supplementary Table 9.

Among the 16 medium- and high-quality MAGs containing reductive *dsrAB*, five acidobacterial MAGs were found to contain a total of six prophages (with genome sizes varying from 14.0 to 58.2 kb; Fig. 3 and Supplementary Table 9), while no viral sequences were detected in the four deltaproteobacterial MAGs. One, seven, and three prophages were found in *T. gabretensis*, *D. vulgaris*, and *D. multivorans*, respectively, which was not in complete agreement with previous findings showing that one, eight, and no prophages were identified in these three species, respectively^33,42,43^. This discrepancy likely resulted from the utilization of a more sensitive and accurate viral prediction method (VirSorter;^44^ see Methods section for details) in this study. Notably, Pfam annotations revealed that 12 out of the 17 prophages identified here harbored at least one virion-associated gene (Supplementary Table 9), suggesting that these prophages still had the genetic potential to complete a lytic cycle^33^.

Nine out of the 17 identified prophages could not be clustered with known isolated viruses or those identified in publicly available microbial genomes or metagenomes using a gene content-based classification (genus-level grouping)^45^, although half of them could be tentatively assigned to the order *Caudovirales* (Supplementary Table 9). Specifically, five acidobacterial prophages formed an exclusive cluster (named Cluster 1), while the remaining two acidobacterial prophages were not closely related to any previously sequenced bacteriophages at the nucleotide level (referred to as singletons^46^; Supplementary Table 9 and Fig. 3). Similarly, among the prophages of *D. vulgaris* and *D. multivorans*, one was affiliated with the *Myoviridae* family, three were clustered exclusively (named Cluster 2), and the remaining six were singletons. In addition, the abundance of the prophages targeting acidobacterial SRMs in our study site was positively correlated with host abundance (*r* = 0.93, *P* < 0.01; Supplementary Fig. 8).

### Roles of viruses in glycoside hydrolysis of SRMs

**Fig. 4:**
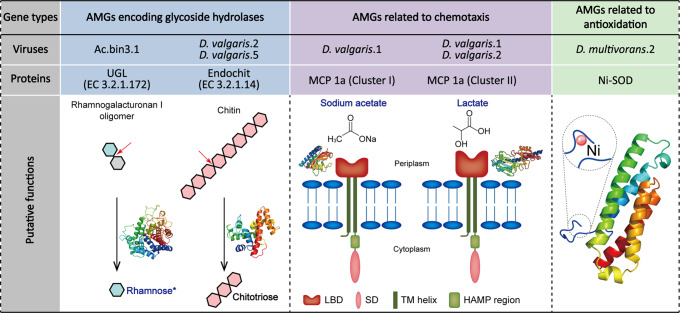
Putative functions of proteins encoded by auxiliary metabolic genes (AMGs) of viruses infecting SRMs. UGL D-4,5-unsaturated β-glucuronyl hydrolase, Endochit endochitinase, MCP methyl-accepting chemotaxis protein, Ni-SOD nickel-containing superoxide dismutase, LBD ligand-binding domain, SD signaling domain, TM transmembrane, HAMP histidine kinase, adenyl cyclase, methyl-accepting chemotaxis protein and phosphatase. Representative substrates of glycoside hydrolases (GHs) encoded by AMGs and the associated simplest products are shown. The cleavage points of the substrates are indicated by red arrows. The computational protein models of GHs, MCPs and Ni-SOD are displayed. Representative ligands of MCPs and the active site of Ni-SOD are shown. *, if the products of GHs or representative ligands of MCPs can be utilized directly by SRMs to reduce sulfate, their names are in blue. Additional details are presented in Supplementary Tables 10 and 11.

Three genes encoding GHs were recovered from viral scaffolds, which were further predicted via three-dimensional protein structural modeling (Fig. 4 and Supplementary Table 10). Among them, one was from the virus infecting Ac.bin3 and encoded D-4,5-unsaturated β-glucuronyl hydrolase (EC 3.2.1.172), which is able to release rhamnose from rhamnogalacturonan I oligomers (a major component of the plant cell wall^47^; Fig. 4). The remaining two genes were identified on the viral scaffolds D. vulgaris.2 and D. vulgaris.5, both of which encoded endochitinase (EC 3.2.1.14). This enzyme can cleave chitin randomly at internal sites, generating soluble low-molecular-mass multimers of *N*-acetyl-d-glucosamine, such as chitotetraose and chitotriose (Fig. 4)^48^.

### Roles of viruses in chemotaxis and antioxidation of SRMs

Three MCPs were encoded by the viral scaffolds D. vulgaris.1 and D. vulgaris.2 (Fig. 4 and Supplementary Table 11). Among them, two belonged to cluster II of class Ia with double cache-like sensor domains, while the other belonged to cluster I of class Ia with a single cache2 domain. According to previous findings^49^ and the ligands confirmed in model protein structures, lactate and C2/C3 carboxylates (e.g., sodium acetate) could be the ligands for these MCPs (Fig. 4). On the other hand, one gene encoding a Ni-containing SOD was identified on the viral scaffold D. multivorans.2 (Fig. 4 and Supplementary Table 11).

### Transcriptomic profile of SRMs

**Fig. 5:**
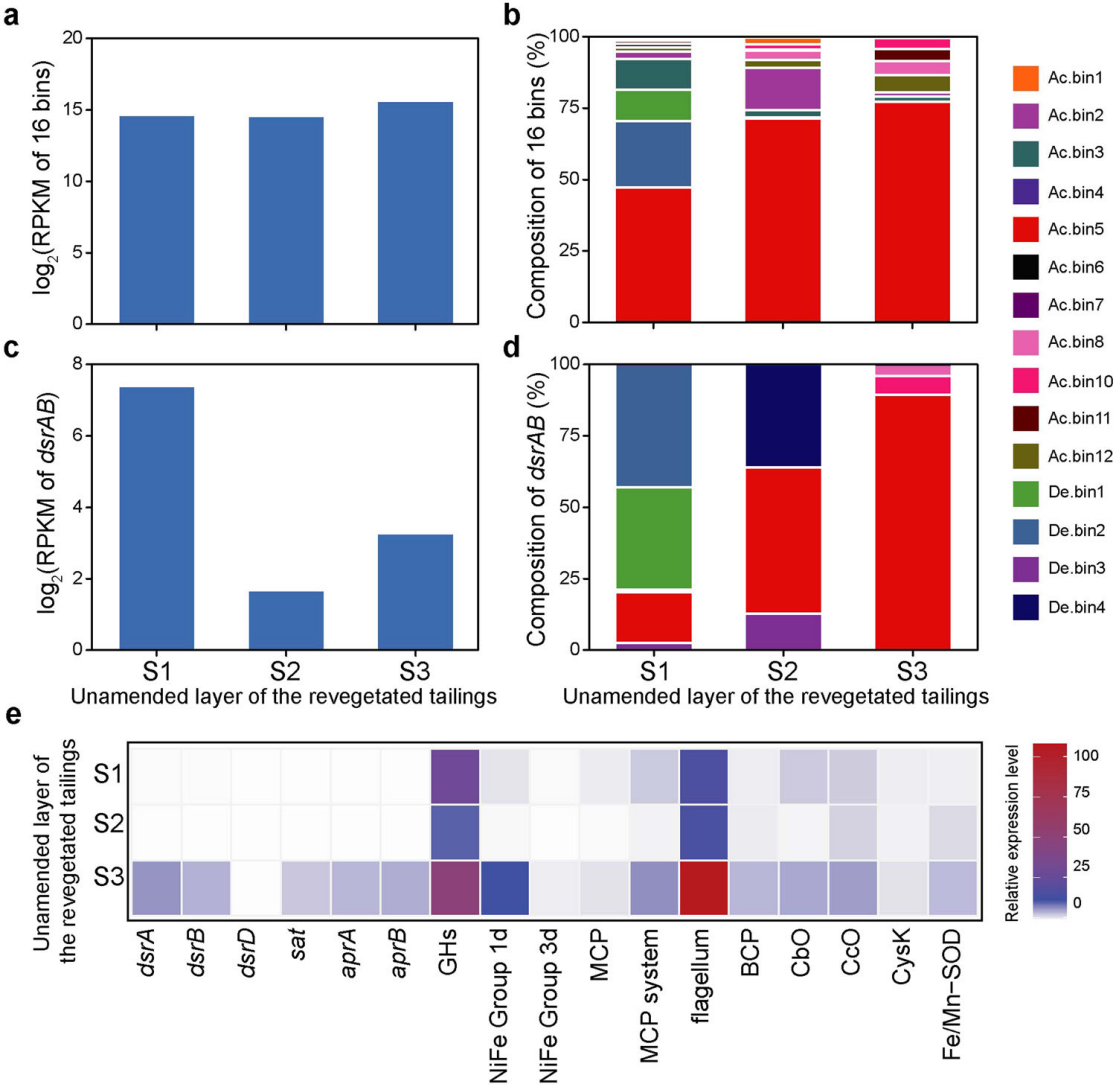
Transcriptional profile of the 16 SRMs reported in this study. **a** Overall transcript abundance of the 16 SRMs. **b** Relative contribution of individual SRMs to the overall transcript abundance of the 16 SRMs. **c** Overall transcript abundance of *dsrAB* of the 16 SRMs. **d** Relative contribution of individual SRMs to the overall transcript abundance of *dsrAB* of the 16 SRMs. **e** Relative expression levels of selected genes of Ac.bin5. Relative expression level was calculated based on the reads per kilobase per million mapped reads (RPKM) of a given selected gene normalized by that of the reference gene *gyrA*. The gene abbreviations are in accordance with those in Fig. 2. The results are based on three soil samples collected from the unamended layer of the revegetated tailings.

Except for Ac.bin9, all the other MAGs reported in this study were transcriptionally active (Fig. 5a, b), together contributing an average of 0.18% of the total mRNA reads in our metatranscriptomes. Remarkably, a majority of the SRM mRNA reads (51–81%; Fig. 5b) were from Ac.bin5. Specifically, 61–83% of the genes encoded by the SRM were expressed. Furthermore, Ac.bin5 was also an important contributor to the *dsrAB* transcripts detected in this study, although its relative contribution varied considerably among samples (Fig. 5c, d). The transcript abundance of Ac.bin5 was positively correlated with soil Eh and negatively correlated with soil Fe^2+^ concentration, while there was a positive relationship between the transcript abundance of *dsrAB* and soil total carbon content (all *r* = 0.99, *P* < 0.03; Supplementary Fig. 9 and Supplementary Table 12).

Compared with the housekeeping gene *gyrA*^50^, the expression levels of *dsrA* and *dsrB* genes of Ac.bin5 were higher, with an average relative expression level (among samples) of 2.5 and 1.8, respectively (Fig. 5e). Note that the relative expression levels of GH genes of Ac.bin5 were extremely high (ranging from 11 to 51; Fig. 5e), with approximately 74–95% of the 129 GH genes of the SRM determined to be active. We found that the genes encoding 1d [NiFe]-hydrogenases, the MCP system (including classes Ia and IVa MCPs), the flagellum system and various antioxidant enzymes (e.g., CbO, Cco, and NPX) of the SRM were also highly expressed, with an average relative expression level ranging from 3.3 to 46 (Fig. 5e).

To further confirm the comparative genomics results, the relative expression levels of selected genes of a representative deltaproteobacterial SRM (i.e., De.bin1, the most dominant high-quality deltaproteobacterial MAG in our metatranscriptomes) were also examined. The genes involving in sulfate reduction and other focal metabolic processes (e.g., glycoside hydrolysis, respiration, denitrification, and antioxidation) were highly expressed as compared to *gyrA*, with average relative expression levels ranging from 1.4 to 76 (Supplementary Fig. 10 and Supplementary Table 13). Interestingly, the average relative expression level of GH genes was 23-times higher in Ac.bin5 than in De.bin1 (*P* < 0.05; Supplementary Fig. 10). As to other genes commonly expressed in Ac.bin5 and De.bin1, their relative expression levels showed no significant differences between the two SRMs (*P* > 0.05). However, the lack of significant differences did not indicate that absolute expression levels of these genes were similar in the two SRMs, because the *gyrA* of Ac.bin5 in the metatranscriptomes was much more abundant than that of De.bin1 (average RPKM: 13.4 *vs* 0.17). Although genes encoding BCP and Fe/Mn-SOD were found in both SRMs, only those in Ac.bin5 were expressed (with an average relative expression level of 2.4 and 2.7, respectively). In addition, transcripts of 1d and 3d [NiFe]-hydrogenases and CcO were detected only in Ac.bin5 (Supplementary Fig. 10), which was consistent with our comparative genomics findings that these genes were possessed by Ac.bin5 but not De.bin1 (Fig. 2).

## Discussion

We performed a genome-centric metagenomic and metatranscriptomic study that was specifically aimed at characterizing SRMs in a constantly oxic/hypoxic terrestrial environment. The recovery of seven high-quality MAGs of SRMs (six acidobacterial and one deltaproteobacterial) provided us with a particular opportunity to explore the metabolic potentials of these SRMs and their viruses, with emphasis on a comparison between *Acidobacteria* and *Deltaproteobacteria*. To this end, the genomes of one aerobic acidobacterial species (i.e., *T. gabretensis*^34^) and two cultured deltaproteobacterial model SRMs with contrasting tolerance to oxygen stress (i.e., the oxygen-tolerant *D. vulgaris* and oxygen-sensitive *D. multivorans*^35^) were also included for comparison. We focused on genes encoding enzymes responsible for energy gain (including GHs and hydrogenases^1,2^) and antioxidation (including antioxidant and chemotaxis enzymes^24^) because the studied mine wasteland was characterized by low soil nutrient levels (especially those of organic compounds that can provide electrons for microbial sulfate reduction^1,2^) and constantly oxic/hypoxic conditions.

### The revegetated mine wasteland fostered new SRM genera

The occurrence of SRMs in *Acidobacteria* was revealed very recently^14,51^. To date, only 13 MAGs of SRMs in *Acidobacteria* (with genome completeness varying from 29.7% to 98.0%) have been reported^14–16,51^. In this context, this study not only doubled the number of such MAGs but also determined the genus-level taxonomic novelty of acidobacterial SRMs (Fig. 1). In addition, a remarkable finding of this study was that one deltaproteobacterial MAG (i.e., De.bin4) represented a genus not included among previously identified SRMs, given that SRMs in *Deltaproteobacteria* were investigated extensively^2^. It is therefore obvious that more work is needed to achieve a ‘complete understanding’ of the taxonomic diversity of SRMs, although recent studies have greatly expanded the diversity of SRMs at the OTU and phylum levels^13,14^. To that end, terrestrial sulfate-rich environments under constantly oxic/hypoxic conditions deserve more attention.

### Acidobacterial SRMs encoded more GHs and oxygen-tolerant hydrogenases than deltaproteobacterial SRMs

Many members of *Acidobacteria* are thought to have the ability to use a wide range of carbohydrates, as they dedicate a large portion of their genomes to carbohydrate metabolism^33^. Furthermore, there was evidence that three DsrAB-encoding acidobacterial MAGs recovered from peatland sediments harbored more GH genes (on average ~105 genes per genome) than not only the other known SRMs but also the majority of non-SRM *Acidobacteria* (~60 genes per genome)^15^. Coincidently, the average number of GH genes in the six high-quality acidobacterial MAGs with reductive *dsrABs* in this study was up to 120 per genome (Supplementary Table 3), which was much greater than that in the three deltaproteobacterial SRMs. This result hinted at an adaptation of these acidobacterial SRMs to the oligotrophic conditions of our study site^31^. Notably, the most prevalent GH family across the six high-quality acidobacterial MAGs (i.e., GH3, Fig. 2) consisted of β-glucosidases (EC 3.2.1.21), β-xylosidases, and *N*-acetylglucosaminidases (EC 3.2.1.52)^52^. These enzymes can liberate glucose and xylose (i.e., growth substrates for SRMs) from plant cell wall-derived oligosaccharides (e.g., cellobiose and xylan)^53^. In contrast, those enzymes belonging to the most abundant GH family across the deltaproteobacterial SRMs (i.e., GH23; Fig. 2) exhibit activity towards peptidoglycan (a main component of the bacterial cell wall) and cannot release monosaccharides as a product^52^. Taken together, these results indicated that acidobacterial SRMs likely predominated over deltaproteobacterial SRMs in oligotrophic environments with plant residues.

Groups 1, 2a, 2b, 3a, 3b, 3c, and 3d of [NiFe]-hydrogenase as well as groups A and B1/B2 of [FeFe]-hydrogenase are known to be involved in the oxidation of H_2_^54^. Two acidobacterial MAGs (i.e., Ac.bin4 and Ac.bin6, Fig. 2) lacked genes encoding such hydrogenases, suggesting their inability to couple sulfate reduction to oxidation of H_2_^1,2^. This characteristic likely placed the SRMs at a disadvantage in the competition for H_2_ with their counterparts^36^ and thereby provides a possible explanation for the fact that these two MAGs occurred in our study site at a lower relative abundance than the other five high-quality MAGs possessing such genes (average 0.031% *vs* 0.045%; Supplementary Table 1). Remarkably, the hydrogenases encoded by the other four high-quality acidobacterial MAGs were oxygen tolerant (i.e., 1d and/or 3d of [NiFe]-hydrogenase, Fig. 2)^36^. This was consistent with a previous finding showing that seven acidobacterial MAGs of SRMs encoded oxygen-tolerant hydrogenases (i.e., 3b and/or 3d of [NiFe]-hydrogenase)^15^. Note, however, that the oxygen-sensitive *D. multivorans* rather than the other two oxygen-tolerant deltaproteobacterial SRMs also encoded an oxygen-tolerant hydrogenase (Fig. 2). These results suggested that the occurrence of oxygen-tolerant hydrogenases in a given SRM could improve its ability to compete for growth substrates in oxic/hypoxic environments but did not necessarily reflect its tolerance to oxygen exposure.

### Acidobacterial SRMs differed from deltaproteobacterial SRMs in terms of oxygen defense mechanisms

MCPs are an indispensable component of the behavior-based antioxidant strategies (including aggregation and aerotaxis) of SRMs^24,37^. Among the three types of MCP genes reported previously to be involved in bacterial aerotaxis (i.e., cluster I of class Ia, class II, and class IVa)^37^, only class II was not recorded here. The two oxygen-tolerant deltaproteobacterial SRMs (i.e., *D. vulgaris* and De.bin1) harbored 17 and 18 genes encoding cluster I of class Ia MCPs, respectively, but no genes for class II and class IVa MCPs (Supplementary Table 7), indicating a predominant role of cluster I of class Ia MCPs in the aerotaxis of oxygen-tolerant deltaproteobacterial SRMs. In fact, classes II and IVa MCPs have not been previously recorded in SRMs^37^. Remarkably, a total of four class IVa MCPs were found in the seven oxygen-tolerant acidobacterial genomes (Supplementary Table 7). Moreover, Ac.bin5 was the only genome encoding two types of MCPs responsible for bacterial aerotaxis among the 10 focal genomes in this study (Supplementary Table 7). This result provided a possible explanation for the dominance of this acidobacterial MAG in transcripts of SRMs in the mine wasteland (Fig. 5b, d), although additional studies are needed to validate the roles of MCPs in oxygen-tolerant acidobacterial SRMs.

SOD and SOR are the main agents acting against superoxide ions in the periplasm and cytoplasm of SRMs, respectively^24^. The widespread distribution of genes encoding SOD across all focal SRM genomes here (Fig. 2) suggested that SOD was necessary for both deltaproteobacterial and acidobacterial SRMs to cope with oxidative stress, irrespective of whether they could tolerate oxygen in the environment. On the other hand, we found that genes encoding SOR were absent in all six high-quality MAGs of acidobacterial SRMs, despite their presence in those of deltaproteobacterial SRMs. A similar pattern was observed for TPX, an enzyme involved in peroxide detoxification in the cytoplasm of SRMs^24^. Therefore, our results indicated a marked difference between deltaproteobacterial and acidobacterial SRMs in terms of enzyme-based antioxidant strategies. That is, acidobacterial SRMs preferred to reduce oxygen to water before generating various types of reactive oxygen species in the cytoplasm, as they tended to have more genes encoding Cco, which can reduce oxygen to water^24^, but fewer genes encoding enzymes involved in detoxification of superoxide and peroxide (including SOR and TPX) than deltaproteobacterial SRMs (Fig. 2 and Supplementary Table 8).

### Viral infection in SRMs was widespread

Viral infection in SRMs was first revealed by Heidelberg et al.^55^, who performed a whole-genome sequence analysis of *D. vulgaris*. This work was subsequently extended by the finding that *Acidobacteria*-, *Candidatus* Aminicenantes-, *Chloroflexi*-, *Deltaproteobacteria*-, *Nitrospirae*-, and *Planctomycetes*-related SRM genomes retrieved from wetland sediments were hosts of prophages^16^. Consistent with these previous studies, we revealed that five medium- to high-quality acidobacterial MAGs from the mine wasteland and the genomes of two cultured deltaproteobacterial model SRMs all contained at least one prophage (Fig. 3). Our results, along with prior findings, indicated that viral infection in SRMs was more widespread than previously thought. Remarkably, the acidobacterial SRM-specific virus/host abundance ratio recorded here approached 1:1 (Supplementary Fig. 8), which was greater than that for the phylum *Acidobacteria* in soils collected worldwide^56^. Thus, viruses infecting the acidobacterial SRMs were inferred to be in a lysogenic phase, which fitted with recent literature that suggests oligotrophic environments, such as the revegetated acidic mine wasteland, could have largely lysogenic viral populations^57^. On the other hand, most of the viruses recorded in this study could not be taxonomically assigned or were not closely related to any known sequenced viruses at the nucleotide level (Fig. 3), supporting the notion that the diversity of environmental viruses is largely unexplored^16^.

### SRM-infecting viruses could contribute to glycoside hydrolysis of their hosts

Viruses are widely believed to have the ability to modulate the S biogeochemical cycle in aquatic environments, as some aquatic viruses were reported to harbor AMGs (i.e., *rdsrA*, *rdsrC*, or *dsrC*) encoding enzymes directly involved in dissimilatory S metabolism^58,59^. In contrast, little is known about the potential roles of viruses in the terrestrial S biogeochemical cycle. Here, we failed to identify viral AMGs encoding enzymes directly responsible for dissimilatory S metabolism. However, we found two viral AMGs encoding enzymes dedicated to the oxidation of organic compounds (i.e., glycoside hydrolysis; Fig. 4 and Supplementary Table 10). These results were remarkable given that no previous studies have documented such metabolic potentials of viruses hosted by SRMs and that the oxidation of organic compounds is coupled to the reduction of sulfate in SRMs^1,2^. In a wider context, a recent analysis of viral community composition and metabolic potentials in mangrove sediments has led to the hypothesis that viral carbohydrate AMGs may provide their hosts with energy for growth by decomposing complex carbohydrates in soil^60^. Interestingly, our results on the viral AMG encoding D-4,5-unsaturated β-glucuronyl hydrolase allowed us to provide a new mechanistic explanation for this hypothesis, as this enzyme can degrade plant cell wall-derived oligosaccharides to rhamnose (Fig. 4), which is accessible directly to SRMs for dissimilatory sulfate reduction^47,53^. With regard to endochitinase encoded by the two prophages in *D. vulgaris*, its products were not reported previously as substrates that could be used by SRMs for sulfate reduction (Fig. 4)^53^. However, some of its products (e.g., chitobiose) can be metabolized further by SRMs for use in cell wall biogenesis^61^. Such synergism should be advantageous for viral hosts in oligotrophic environments, shedding some light on the ‘black box’ of soil virus–host interactions^57^.

### SRM-infecting viruses could participate in chemotaxis and antioxidation of their hosts

There was only one previous study that documented viral AMGs encoding MCPs^62^. We extended this work by identifying a specific host of such AMGs (i.e., *D. vulgaris*; Fig. 4). Moreover, our in-depth analysis of the viral MCPs recorded here revealed that they possessed an extracellular ligand-binding domain for C2/C3 carboxylates or alanine/lactate (Fig. 4 and Supplementary Table 11). Notably, most of the targeted ligands of these MCPs are growth substrates used by SRMs for sulfate reduction^53^. This characteristic of these MCPs could endow the host with a survival advantage in oligotrophic environments by helping the host sense its growth substrates. With regard to the viral AMG encoding the antioxidant enzyme nickel-containing superoxide dismutase (Ni-SOD; Fig. 4), none of its counterparts (i.e., the AMGs encoding various kinds of SODs) were reported previously. However, in a wider context, there were two prior reports on viral AMGs involved in antioxidation of their host. The first one showed that three AMGs (i.e., *yfdK*, *yfdO,* and *yfdS*) of prophages in *Escherichia coli* were able to enhance their host’s ability to resist oxidative stress^63^. The second showed that some prophages of oceanic cyanobacteria harbored genes encoding a peroxidase, which could improve the hosts’ antioxidative ability^64^. Thus, it is interesting to explore the generality of the involvement of viruses in chemotaxis and antioxidation of their hosts.

### A new acidobacterial SRM harboring a prophage was very active

Many SRMs are known to be merely present as dormant cells under stressful environmental conditions^28^. To our knowledge, there was only one prior study showing clear evidence for the activity of SRMs in constantly oxic/hypoxic terrestrial environments^65^. In that study, SRM enumeration and geochemistry were used in combination with S isotopic analysis to infer the activity of SRMs in mine tailings. The present study provides transcriptomic evidence for the activity of SRMs (especially at the species level) in such environments. Our finding that a new acidobacterial SRM (i.e., Ac.bin5) containing a prophage dominated the transcripts of the 16 SRMs reported in this study (Fig. 5b, d) was reasonable given that, even in anoxic environments, only a minority of SRMs were metabolically active^15^.

We obtained several clues to understand the dominance of Ac.bin5. First, transcripts of oxygen-tolerant 1d and 3d [NiFe]-hydrogenases were detected in Ac.bin5 but not in a less active SRM (i.e., De.bin1; Supplementary Fig. 10). Second, Ac.bin5 not only encoded a complete MCP system (with two types of MCPs involved in bacterial aerotaxis) and a complete flagellum system (Fig. 2 and Supplementary Tables 6, 7) but also expressed the relevant genes (Fig. 5e), indicating that it was able to tolerate oxygen in the environment via an MCP-dependent behavior-based antioxidant strategy^24^. Third, Ac.bin5 harbored the greatest number of genes encoding Cco (a high-affinity oxygen reductase^66^) among the 10 focal genomes (Fig. 2 and Supplementary Table 8), and these genes were expressed at a level comparable to that of Cbo (another high-affinity oxygen reductase; Fig. 5e). This pattern was very different from that of *D. vulgaris*, in which the expression level of Cco genes under oxygen stress conditions was much lower than that of Cbo^67^. These findings provided further evidence for our speculation mentioned above, i.e., that acidobacterial SRMs tended to reduce oxygen to water before generating various types of reactive oxygen species in the cytoplasm. Fourth, the expression of those genes encoding enzymes responsible for scavenging superoxides and peroxides (e.g., Fe/Mn-SOD and NPX; Fig. 5e)^24^, along with MCP, Cco and Cbo, possibly constituted a multilayer and efficient antioxidant system for Ac.bin5. This possibility was supported by our observation that: (1) the expression level of Ac.bin5 was positively related to soil Eh but negatively related to soil Fe^2+^ concentration (Supplementary Fig. 9); and (2) De.bin1 was found not to transcribe genes encoding BCP, Fe/Mn-SOD and Cco (Supplementary Fig. 10). Finally, Ac.bin5 contained the second largest number of GH genes (Fig. 2 and Supplementary Table 3) and the relative expression level of GH genes was significantly higher in Ac.bin5 than in De.bin1 (Supplementary Fig. 10). These results indicated that high-level expression of GH genes could help Ac.bin5 compete for organic matter in oligotrophic environments (Supplementary Table 12)^33^. Despite this, the functioning of Ac.bin5 was likely inhibited by low carbon availability in the mine wasteland, as indicated by a positive relationship between the *dsrAB* expression level of this SRM and soil total carbon content (Supplementary Fig. 9).

Note also that the expression level of *dsrAB* was not significantly related to the concentrations of the main S compounds in the soil (sulfate and sulfide; *P* > 0.05, Supplementary Fig. 9). A similar pattern was observed for those genes responsible for the oxidation of sulfide (e.g., *soxXAYZB*, Supplementary Fig. 11). These results were consistent with the prior notion that interactions between S reducers and oxidizers may lead to a scenario in which end products or reaction intermediates of the S cycle remain in steady state, but this does not necessarily represent a lack of microbial S cycling^17^. Unfortunately, we found little transcriptomic evidence for the potential roles of SRM-infecting viruses discussed above, consistent with the result of another analysis wherein we used a newly released prophage activity estimator^68^ to show that the prophages recorded in this study were inactive at the sampling time point.

In conclusion, we recovered 16 reductive *dsrAB-*containing MAGs affiliated with *Acidobacteria* and *Deltaproteobacteria* from a revegetated sulfate-rich mine tailings pond under constantly oxic/hypoxic conditions. Among them, five were shown to represent three new SRM genera. Comparative genomics and metatranscriptomics showed that the acidobacterial and deltaproteobacterial SRMs employed different survival strategies for living in the mine wasteland. These findings supported our hypothesis. More importantly, they not only improve our understanding of the diversity and metabolic potentials of SRMs in terrestrial environments under constantly oxic/hypoxic conditions but also provide metatranscriptomic evidence for their activity in situ. Additionally, this study sheds some light on the putative roles of soil viruses in the terrestrial S biogeochemical cycle.

## Methods

### Study site and soil sampling

We selected a revegetated acidic mine wasteland located in southern China (29°40′52″N, 115°49′21″E) as our study site. Briefly, this site was revegetated in the spring of 2013 and consisted of three different habitats: an amended layer of revegetated tailings (0–10 cm, ALRT), an unamended layer of revegetated tailings (11–20 cm, ULRT) and unrevegetated tailings (UT). These habitats were rich in sulfate (1.80–25.9 g SO_4_^2−^ kg^−1^ dry soil) and were under constantly oxic/hypoxic conditions (as indicated by a soil Eh range of ~180–680 mV)^31^. Three independent soil samples for metagenomic analysis were collected from each of these habitats in July 2016 and 2017. More details on the study site, revegetation scheme, soil sampling and soil physicochemical properties are presented elsewhere^31^. To investigate microbial metabolic activities in situ, three additional soil samples were collected from ULRT for metatranscriptomic analysis. The soil samples were preserved in liquid nitrogen until arrival at our laboratory and were stored at −80 °C until further processing.

### DNA extraction and sequence processing

The procedures used for DNA extraction, metagenomic sequencing, and data processing (including assembly, binning, refinement, genome completion estimates, gene prediction, etc.) were described in detail elsewhere^31^. Briefly, soil DNA was extracted using PowerSoil DNA isolation kit (Mobio Laboratories Inc., USA) with modification. The purified DNA was sequenced (250 or 150 bp paired-end reads) using an Illumina MiSeq sequencer (Illumina, USA). Raw sequencing reads were processed by eliminating duplicated reads, reads with ≥5 “N” and reads with quality score <30. The remaining high-quality reads from each metagenomics sample were assembled using SPAdes v3.9.0^69^. The assembled scaffolds from each sample were binned using MetaBAT v0.30.3 with default parameters^70^. The refinement of MAGs were performed by RefineM v0.0.14 first and then by manual examination^71^. For the evaluation of contamination and the completeness of MAGs, CheckM v1.0.4 was used^72^. Gene prediction of these MAGs was conducted using Prodigal v2.6.3^73^.

### Retrieval of key genes involved in dissimilatory S metabolism

The genome-specific metabolic potential for sulfate/sulfite reduction was determined as follows. All predicted open reading frames (ORFs) in a given MAG were searched against the eggNOG^74^ and KEGG^75^ databases using Diamond^76^ and against HMM profiles using InterProScan^77^. Then, the key sulfate reduction/S oxidation genes (*dsrAB*, *dsrD*, *dsrT*, *dsrMKJOP*, *aprAB*, *sat*, and *dsrEFH*) in the MAGs were identified based on conserved domain hits elaborated by Anantharaman et al.^14^.

### Phylogenetic analysis of DsrAB protein

A total of 214 DsrAB sequences (Supplementary Fig. 1), including those from both SRMs and non-SRMs reported previously^14,78,79^, were used for phylogenetic analysis, which could help to distinguish reductive and oxidative type DsrAB. The DsrAB sequences were aligned using MUSCLE^80^ with default parameters. The alignments were then filtered by TrimAL^81^ with the parameters -gt = 0.95 and -cons = 50. The concatenated DsrAB tree was constructed using RAxML^82^ with the parameters set as -f a -m PROTGAMMAIJTT –p 12345 –x 12345 -# 100. The Newick files with the best tree topology were uploaded to the Interactive Tree of Life (iTOL) online interface^83^ for visualization and formatting.

### Sequence alignment of DsrD and DsrT proteins

The DsrD and DsrT protein sequences identified in the MAGs reported in this study were aligned along with the reference sequences respectively, using ClustalW with slow/accurate setting parameters (https://www.genome.jp/tools-bin/clustalw). The alignments were manually corrected and later visualized by ESPript 3.0^84^. The conserved residues were highlighted.

### Taxonomic classification of MAGs containing reductive *dsrAB*

Sixteen MAGs retrieved in our study harbored reductive *dsrAB* sequences (Supplementary Table 1). The direction of dissimilatory S metabolism for each MAG was determined according to the common patterns elaborated by Anantharaman et al.^14^. Taxonomic assignment of the 16 MAGs was inferred from the phylogenetic tree constructed with the reference genomes using GTDB-Tk^85^. For acidobacterial subdivision-level classification, the 12 MAGs of *Acidobacteria* recovered in our study were used for phylogenetic analysis with published reference genomes spanning subdivisions 1, 3, 4, 6, 8, and 23^15,33^. One deltaproteobacterial MAG from the *Syntrophobacteraceae* family without genus-level classification was used for phylogenetic tree construction with public reference genomes from *Syntrophobacteraceae* downloaded from GTDB-Tk. The maximum-likelihood phylogenetic trees were constructed based on a concatenated dataset of 400 universally conserved marker proteins using PhyloPhlAn^86^ and visualized using iTOL.

### Calculation of the average AAI

The AAI values between five genomes from *Syntrophobacteraceae* family were calculated by AAI calculator (http://enve-omics.ce.gatech.edu/aai/index) with default parameters. The reciprocal best hits (two-way AAI) between two genomic datasets of proteins were used for further comparisons.

### Calculation of relative abundances of MAGs

The relative abundances of the 16 MAGs were calculated based on the methods described elsewhere^31^. Briefly, the high-quality reads from each genomic dataset were mapped to all of the dereplicated MAGs using BBMap with the parameters *k* = 14, minid = 0.97, and build = 1. The coverage of a given MAG was calculated as the average scaffold coverage, and each scaffold was weighed by its length in base pairs. Then, the coverage of each MAG divided by the total coverage of all MAGs in each sample was considered its relative abundance.

### Selection of genomes for metabolic potential analysis

Seven high-quality MAGs containing reductive *dsrAB* were chosen for further metabolic potential annotation, including six *Acidobacteria* (i.e., Ac.bin1–Ac.bin6) and one *Deltaproteobacteria* (i.e., De.bin1). The genome sequences of two cultured model SRMs (i.e., *D. vulgaris* Hildenborough (oxygen-tolerant) and *D. multivorans* DSM 2059 (oxygen-sensitive)^35^) and one cultured non-SRM species of *Acidobacteria* (i.e., *T. gabretensis* S55 isolated from oxic forest soil^34^), which was shown to be the species most closely related to eight out of the 12 acidobacterial MAGs in this study (Supplementary Fig. 2), were also selected for comparative genomic analysis^43,55^.

### Identification of carbohydrate-active enzymes

To identify carbohydrate-active enzyme genes, all predicted ORFs in the 10 selected genomes were searched against the dbCAN2 meta server^87^ with default parameters: HMMER (*E*-value < 1e-15, coverage > 0.35), Diamond (evaluation < 1e-102) and Hotpep (frequency > 2.6, hits > 6). Those identified by at least two tools were kept for further classification of GH families using an in-house Perl script.

### Identification of hydrogenases

For identification of hydrogenases, HMM searches were performed by searching all predicted ORFs in the 10 selected genomes. Briefly, the individual HMM profiles for [NiFe]-hydrogenases from groups 1a–1h, 2a–2d, 3a–3d, and 4a–4e, [FeFe]-hydrogenases from groups A1–A4, B and C, and Fe hydrogenase were generated using the reference sequences retrieved from a previous study^36^. The reference sequences were aligned using MUSCLE with default parameters, and then, the alignment was converted to Stockholm format, and databases were built using hmmscan^88^. The noise cut-offs for individual HMM profiles were determined by manual inspection. Protein sequences that showed the best hit with the HMM profiles with (1) bit scores greater than the calibrated threshold and (2) over 90% sequence coverage were retained.

### Identification of proteins involved in respiration

All predicted ORFs in the 10 selected genomes were searched for proteins involved in four respiratory complexes based on the eggNOG annotation results (protein IDs are provided in Supplementary Table 5), including NADH dehydrogenase, succinate dehydrogenase, quinol-cytochrome-c reductase, terminal oxidase, and ATP synthase^15^.

### Identification of proteins involved in chemotaxis and oxidative stress

In addition to MCPs, the central components of the bacterial chemotaxis system include CheA, CheB, CheR CheW, and CheY^38^. MCPs in the 10 selected genomes were identified by Pfam annotation hits to PF00015, while the other central protein sequences were identified by KEGG annotation hits and were further confirmed based on eggNOG annotations. Classification of MCPs was performed according to the ligand binding region and membrane topology^37^. Proteins involved in the core flagellum^40^ and type IV pilus^41^ systems were identified by KEGG annotation hits and were further confirmed by eggNOG annotation results. The antioxidative enzymes analyzed in this study were selected based on two previous reviews^24,89^ and were identified by KEGG, eggNOG and InterPro annotations.

### Recovering and annotating viral scaffolds

VirSorter^44^ was used to recover viral scaffolds from the 16 MAGs as well as the genomes of all three cultured model species mentioned above. Only scaffolds from VirSorter categories 1, 2, 4, and 5 (categories 4 and 5 represent the provirus equivalents of categories 1 and 2) were retained. Specifically, viral scaffolds from categories 1 and 4 contain sequences similar to known viruses, and those from categories 2 and 5 contain viral hallmark genes and/or are enriched with viral or non-*Caudovirales* genes and have at least one other virus-like metric^44,56^. For scaffolds with predicted proviruses, only predicted proviral regions were retained. To taxonomically classify the viral scaffolds, a gene content-based network analysis was performed to cluster viral scaffolds into viral clusters at approximately the genus level using vConTACT2 with the ProkaryoticViralRefSeq94 database^45^. The ORFs in the viral scaffolds were predicted with MetaProdigal. Viral signature proteins such as terminase, integrase, capsid, and tail were identified by Pfam hits. Viral sequences that encode tail genes could be tentatively assigned to the order *Caudovirales*.

### Estimation of viral and host abundances

The abundance of a given virus and that of its host were calculated as the normalized mean coverage depth based on the methods described elsewhere^56^. Briefly, the high-quality reads from each metagenomic dataset were mapped to all of the dereplicated viral scaffolds or dereplicated MAGs using BBMap with the parameters *k* = 14, minid = 0.97, and build = 1. The viral and host abundances were pulled from the BBMap mapping coverage output and normalized to the number of metagenomic reads in each sample.

### Identification of viral AMGs

To examine the potential roles of the identified viruses in S biogeochemistry, we assessed whether they contained AMGs. The predicted viral proteins were searched against the dbCAN2 meta server^87^ and with HMM profiles using InterProScan^77^ as mentioned above. For GHs, MCPs, and Ni-SOD encoded by AMGs identified in this study, the protein sequences were structurally modeled using PHYRE2^90^ in normal modeling mode to confirm and further resolve functional predictions.

### RNA extraction and sequencing

Total cellular RNA was extracted using the RNeasy PowerSoil Total RNA kit (QIAGEN, USA) according to the manufacturer’s instructions. Total RNA was transported to the Magigene Company (Guangzhou, China) on dry ice for subsequent rRNA subtraction, cDNA synthesis, library construction, and sequencing with an Illumina NovaSeq platform (paired-end 150-bp mode).

### Metatranscriptomic analysis

Raw reads were filtered by fastp^91^ with the parameters --cut_mean_quality 20 and -l 50. The rRNA sequences from prokaryotes and eukaryotes were removed by SortMeRNA^92^ with default parameters. Subsequently, the remaining reads were mapped to the genes predicted from metagenomic assemblies using BBMap with the parameters *k* = 14, minid = 0.97, and build = 1. The detailed information of metatranscriptomic datasets was provided in Supplementary Table 14. The expression level for each gene in each sample was normalized to reads per kilobase per million mapped reads (RPKM) values. The transcript abundance of each MAG in a given sample was calculated as the RPKM sum of all transcripts within that MAG. Similarly, the transcript abundance of a given gene from the 16 SRM-related MAGs reported in this study was calculated as the RPKM sum of all transcripts assigned to that gene from these MAGs. To compare relative gene expression levels between different SRMs, the housekeeping gene *gyrA* was chosen as the reference gene^50^. The relative expression level of the selected genes was calculated as RPKM values of the genes normalized by that of the reference gene *gyrA*.

### Reporting summary

Further information on research design is available in the Nature Research Reporting Summary linked to this article.

## Supplementary information

Supplementary Information

Reporting Summary

## Data Availability

The metagenomic and metatranscriptomic data analyzed in this study were deposited at EMBL under accession numbers PRJEB31441 and PRJEB42658, and the MAGs reported in this study have been deposited in GenBank under accession numbers SAMN15699825 and SAMN15808056–70.

## Code availability

All software used is described in the ‘Methods’ and the code used for analysis that support findings of this study are freely available online (https://github.com/liangjl-git/SRM_scripts).
