# Supplementary material for: Metagenomic and metatranscriptomic insights into sulfate-reducing bacteria in a revegetated acidic mine wasteland
Source: NPJ Biofilms Microbiomes. 2022 Sep 6;8:71. doi: 10.1038/s41522-022-00333-9 (PMC9448743; doi:10.1038/s41522-022-00333-9)
Supplement: Supplementary file 1 — Supplementary Information [file 41522_2022_333_MOESM1_ESM.pdf]

Supplementary information for

Metagenomic and metatranscriptomic insights into sulfate-reducing bacteria in a revegetated acidic mine wasteland

Jin-tian Li[1,2,†], Pu Jia[1,†], Xiao-juan Wang[1], Shu-ning Ou[1], Tao-tao Yang[2], Shi-wei Feng[1], Jing-li Lu[1], Zhou Fang[1], Jun Liu[2], Bin Liao[2], Wen-sheng Shu[1], & Jie-Liang Liang[1,*]

[1]Institute of Ecological Science, Guangzhou Key Laboratory of Subtropical Biodiversity and Biomonitoring, Guangdong Provincial Key Laboratory of Biotechnology for Plant Development, School of Life Sciences, South China Normal University, Guangzhou 510631, PR China

[2]School of Life Sciences, Sun Yat-sen University, Guangzhou 510275, PR China

[†]These authors contributed equally to this work.

*Corresponding author:

School of Life Sciences, South China Normal University, Guangzhou 510631, PR China

E-mail: Jie-Liang Liang (liangjl@m.scnu.edu.cn)

Tel.: +86 20 85211850; Fax: +86 20 85211850

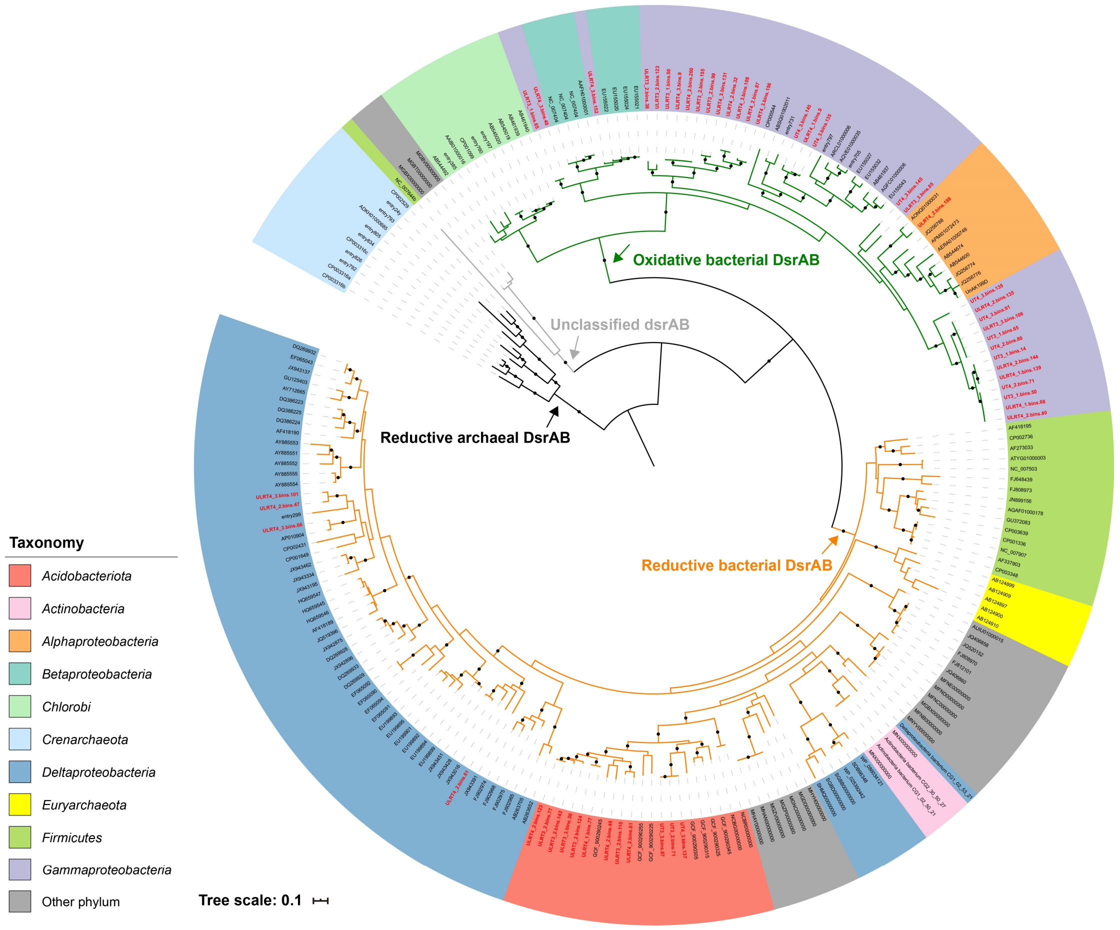

Supplementary figure 1. Phylogenic analysis of dissimilatory sulfite reductases DsrAB. Concatenated DsrAB protein sequences identified in this study are marked in bold red. Assignment of oxidative/reductive, bacterial/archaeal type DsrAB was according to previous studies[1, 2]. Bootstrap values were based on 100 replicates, and only bootstrap values higher than 70% are shown with black circles. Tree scale bar = 0.1.

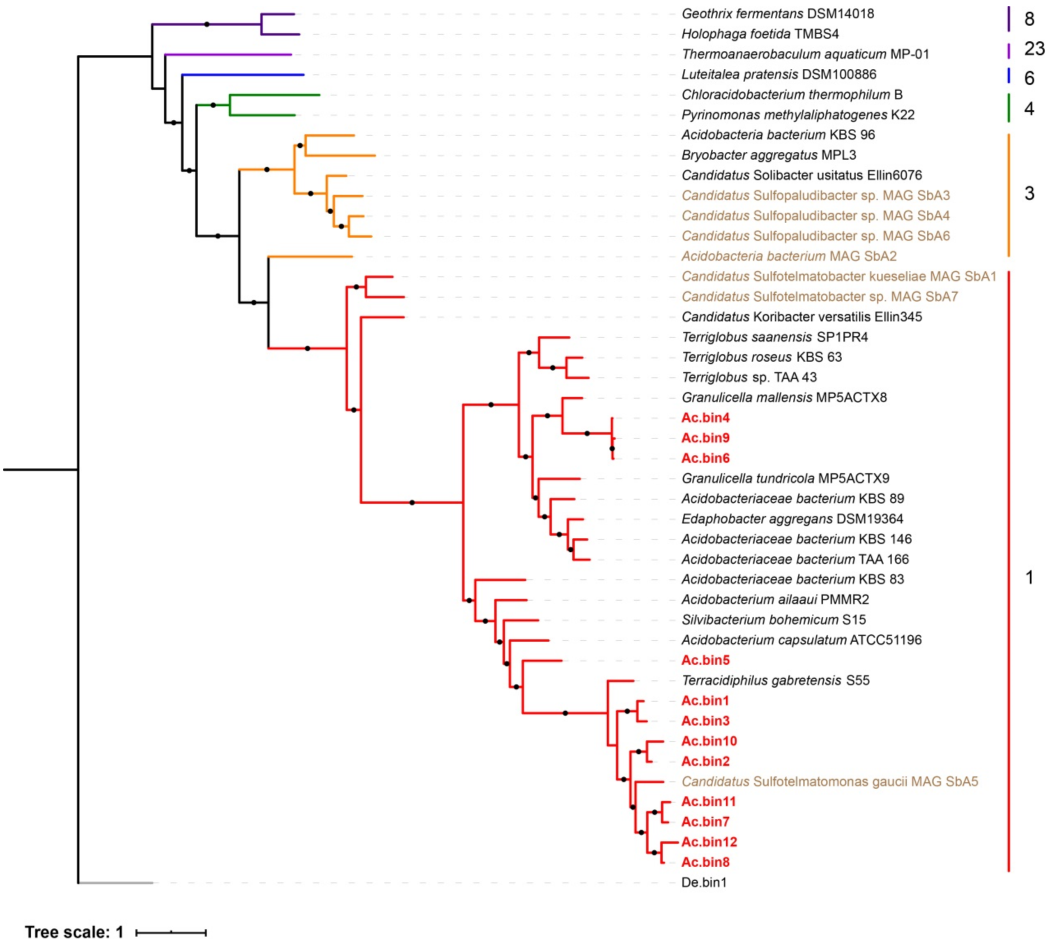

Supplementary figure 2. Phylogenic analysis of the genomes from *Acidobacteria* phylum. The maximum-likelihood phylogenetic tree was constructed based on a concatenated dataset of 400 universally conserved marker proteins using PhyloPhlAn. Subdivisions are indicated to the right of the tree. The 12 metagenome-assembled genomes (MAGs) harboring sequences of *dsrAB* genes reported in this study are marked in bold red. The 7 MAGs previously reported to have a dissimilatory sulfur metabolism potential are marked in brown. Bootstrap values were based on 100 replicates, and percentages higher than 70% are shown with black circles. The MAG (De.bin1) from *Deltaproteobacteria* was used as an outgroup. Tree scale bar = 1.

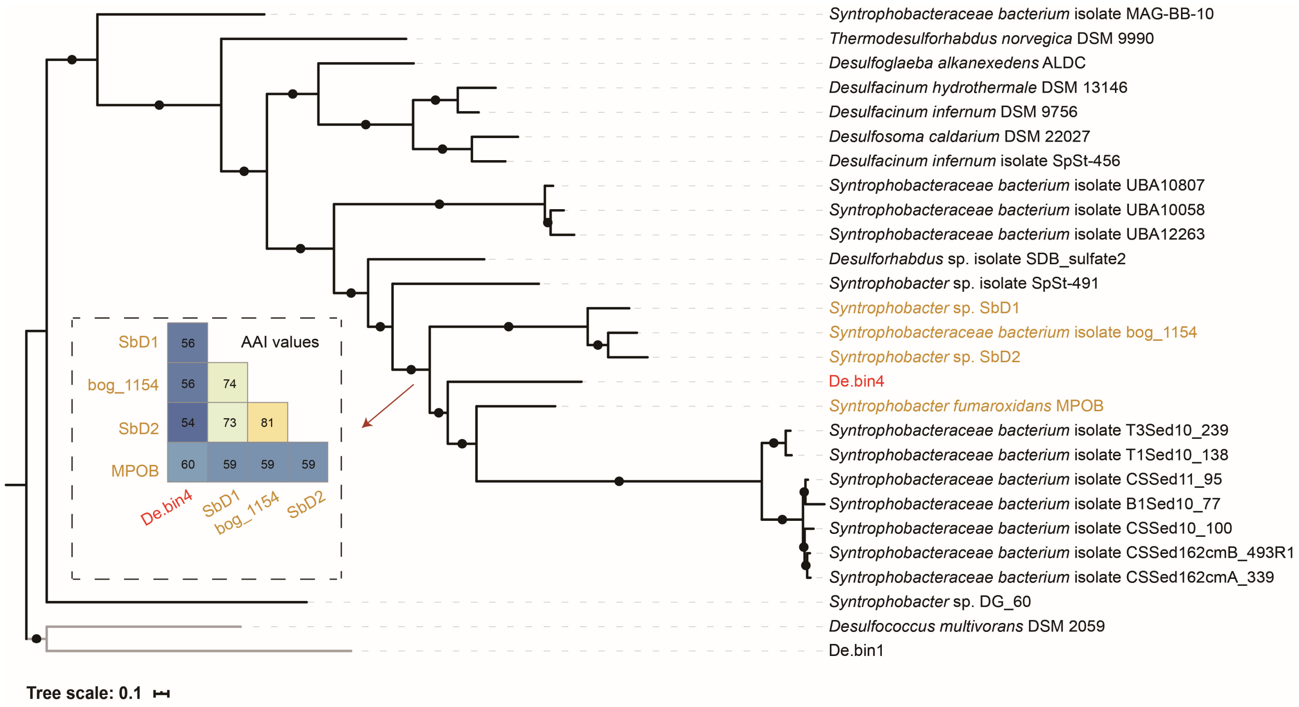

Supplementary figure 3. Phylogenic analysis of the genomes from *Syntrophobacteraceae* family. The maximum-likelihood phylogenetic tree was constructed using PhyloPhlAn. The *dsrAB*-containing MAG reported in this study (i.e. De.bin4) is marked in bold red. The four reference genomes of the two closest genera to the MAG are marked in brown. The average amino acid identity (AAI) values between the 5 closest genomes are shown in the inset. Bootstrap values were based on 100 replicates, and percentages higher than 70% are shown with black circles. Two genomes (De.bin1 and *Desulfococcus multivorans* DSM 2059) from *Deltaproteobacteria* were used as the outgroup. Tree scale bar = 0.1.

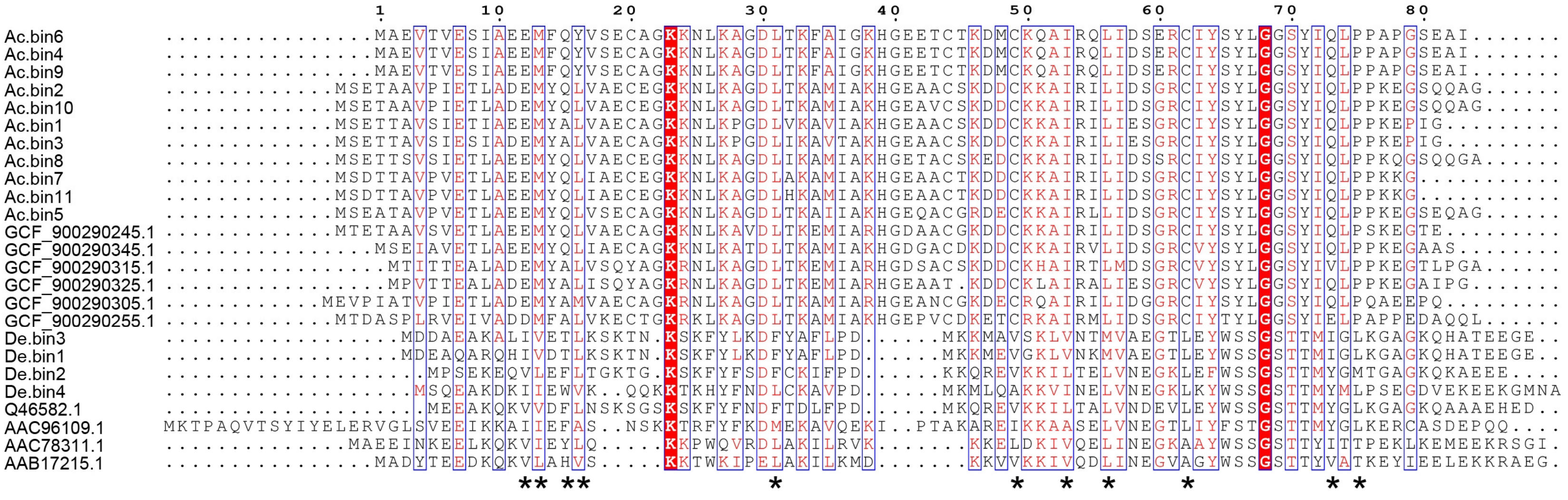

Supplementary figure 4. Sequence alignment of DsrD proteins identified in this study with reference sequences. Positions of conserved, hydrophobic residues in the DsrD family are marked with asterisks[3]. Highly conserved residues of the same type are highlighted in red background.

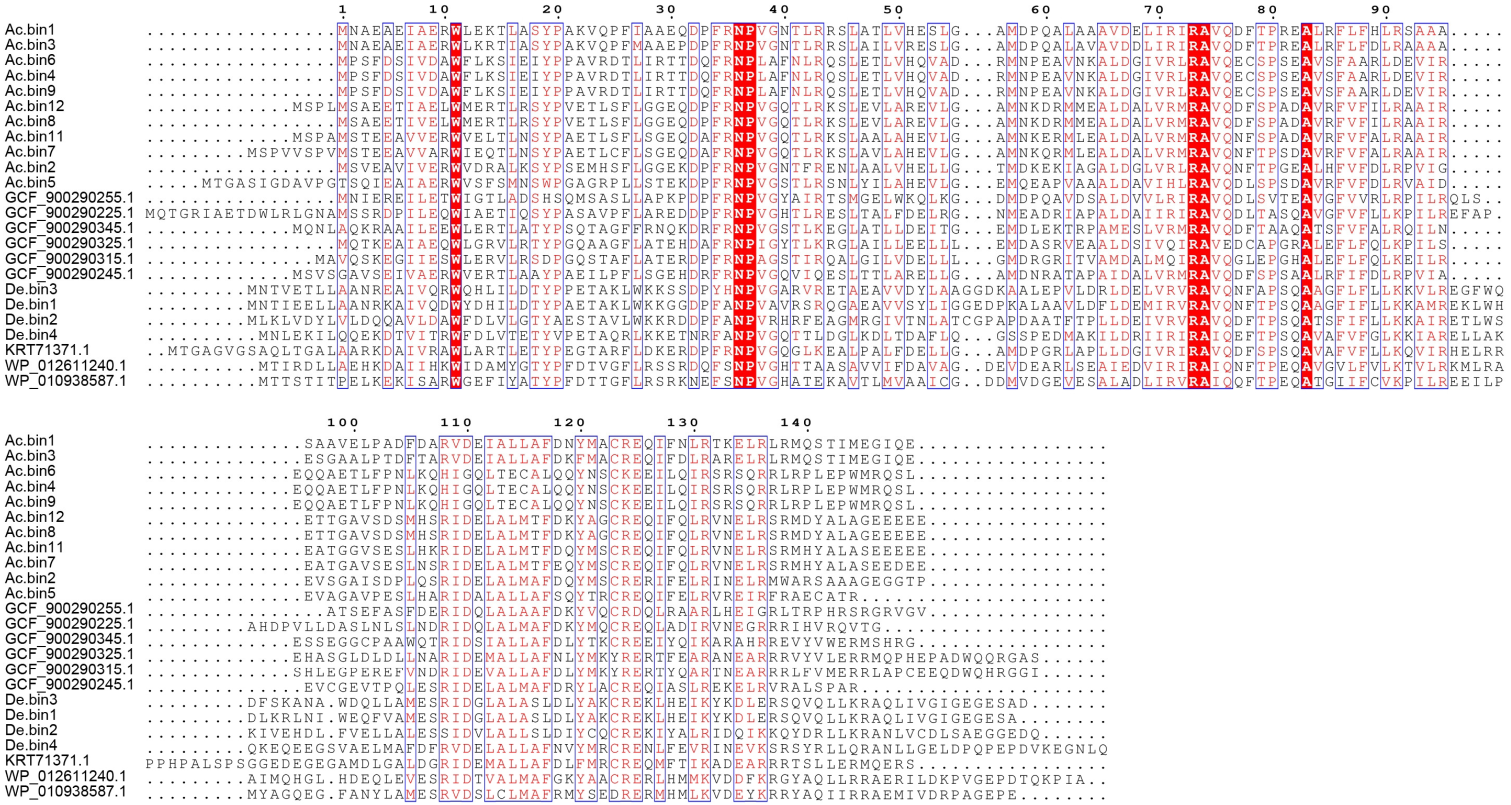

Supplementary figure 5. Sequence alignment of DsrT proteins identified in this study with reference sequences. Highly conserved residues of the same type are highlighted in red background.

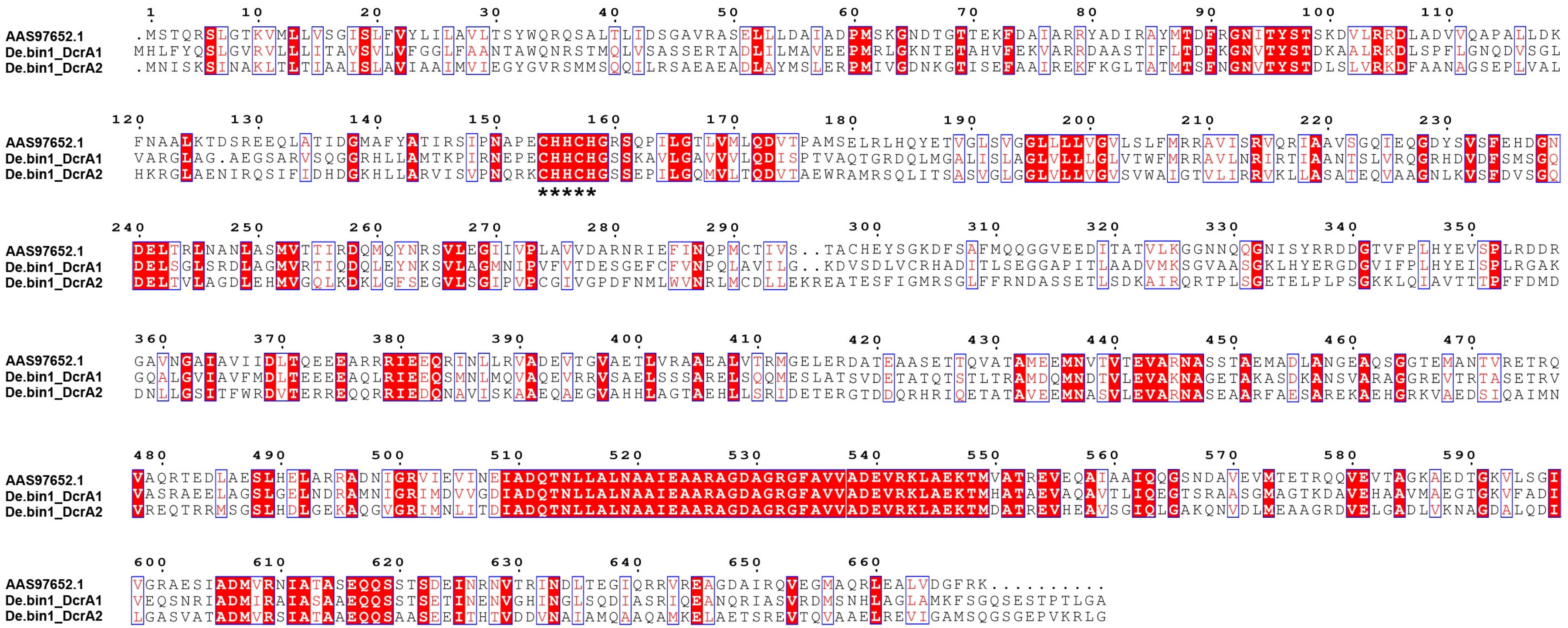

Supplementary figure 6. Sequence alignment of DcrA proteins identified in this study with reference sequence identified in *Desulfovibrio vulgaris* str. Hildenborough. Specific sequences, CHHCH, corresponding to a consensus c-type heme-binding site are marked with asterisks[4]. Highly conserved residues of the same type are highlighted in red background.

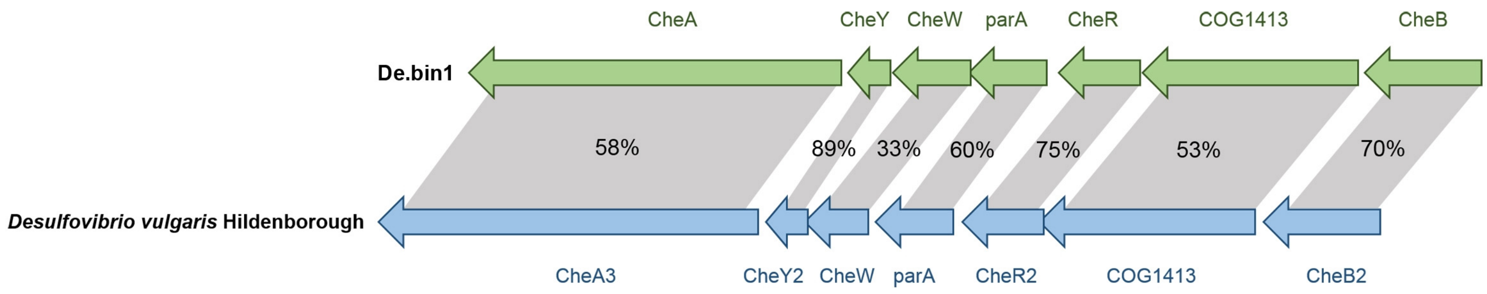

62

63 **Supplementary figure 7.** Operons encoding the *cheA* chemotaxis gene in De.bin1 and *D. vulgaris*[5]. Arrows represented the coding regions. The

64 percentages in grey shadings indicate sequence similarities.

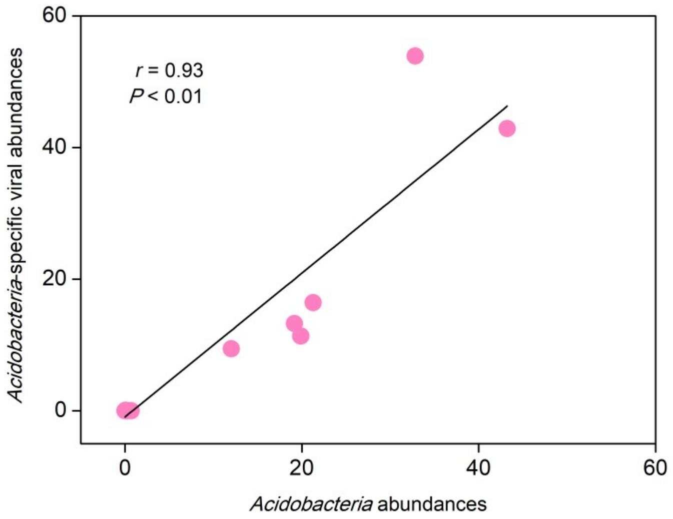

Supplementary figure 8. Virus/host abundance ratio for *Acidobacteria* with a dissimilatory sulfur metabolism. *Acidobacteria* abundance and the abundance of their viruses (both calculated as the mean coverage depth from reads mapping, normalized by the number of reads in each metagenomic dataset) are plotted for each sample. Pearson correlation analysis was used to correlate the host and viral abundance.

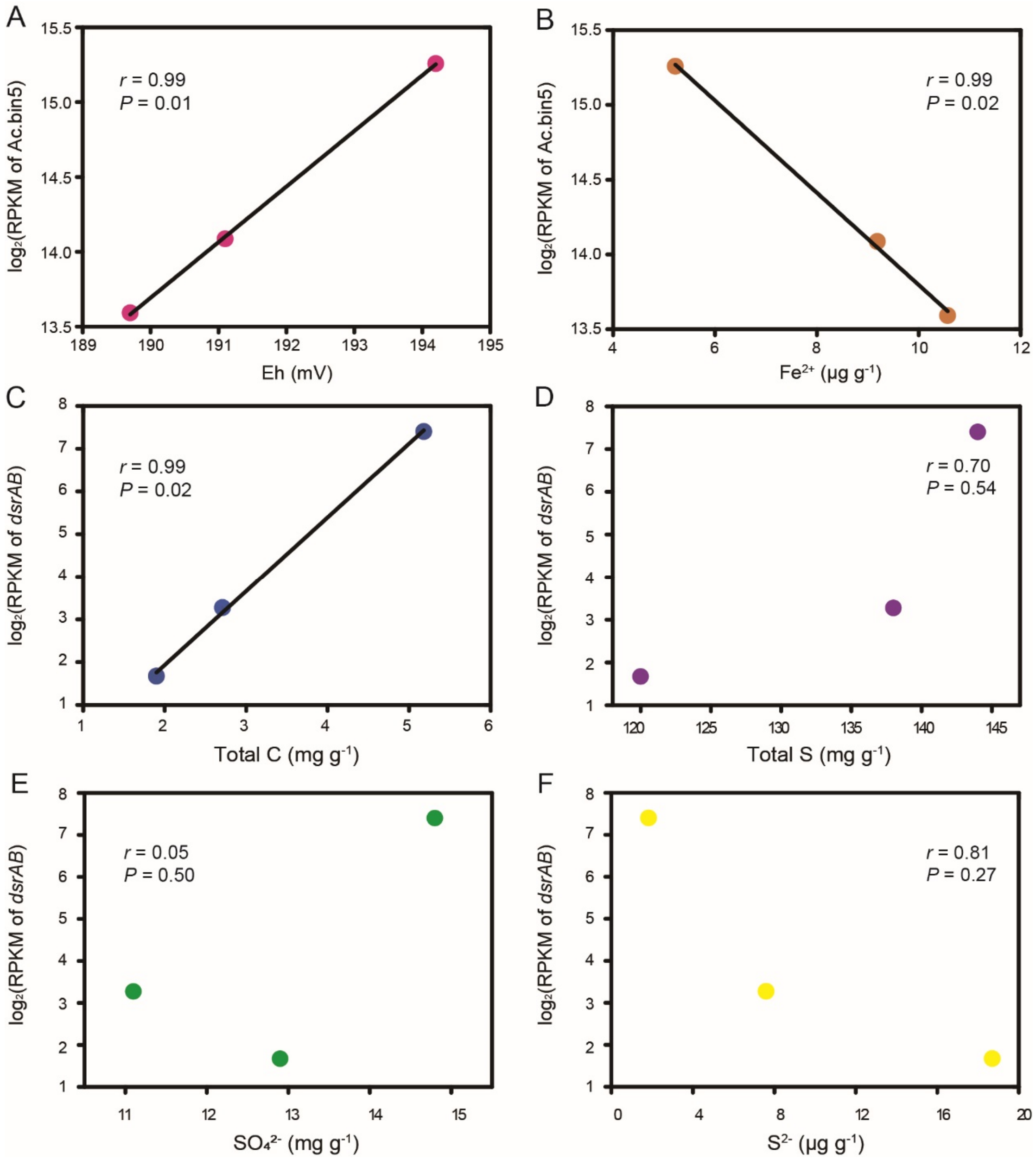

Supplementary figure 9. Correlations between transcript abundance of Ac.bin5 and soil Eh (a) and ferrous iron concentrations (b), and correlations between transcript abundance of *dsrAB* and concentrations of soil total carbon (c), total sulfur (d), sulfate (e) and sulfide (f). Results are based on Pearson correlation analysis.

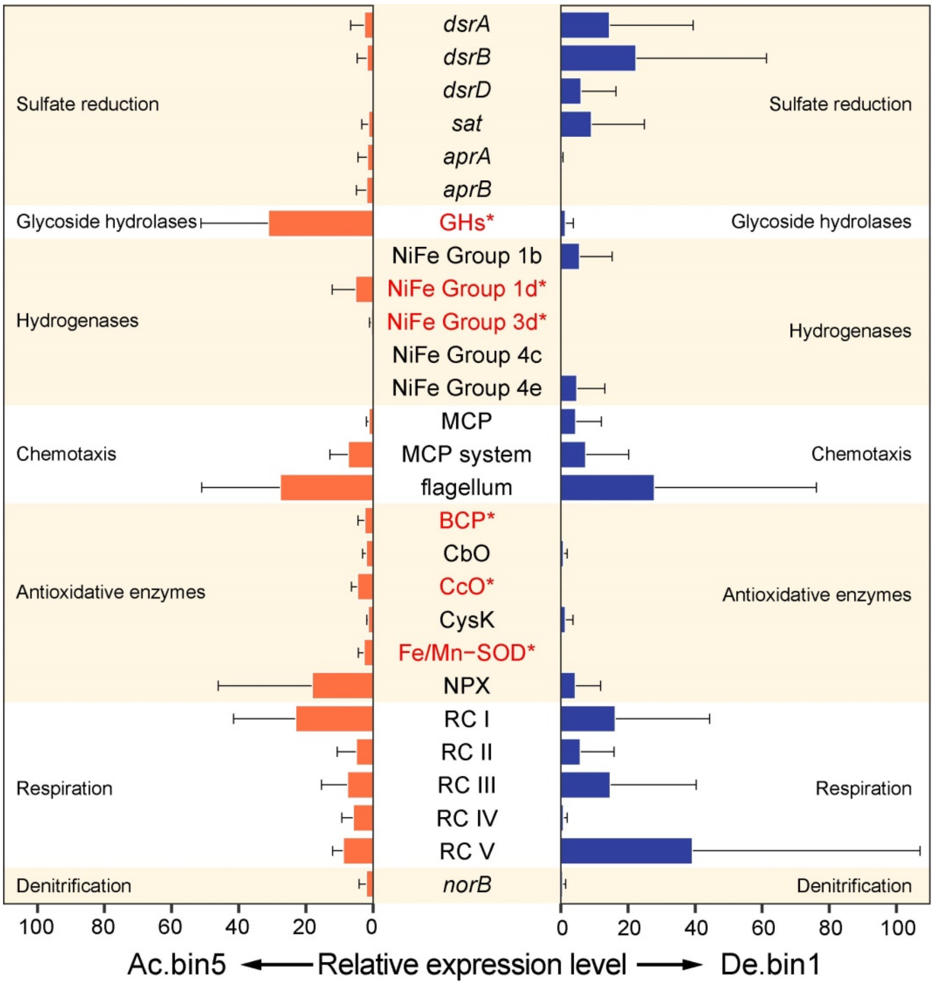

Supplementary figure 10. Comparison of Ac.bin5 and De.bin1 on relative expression levels of genes involving in selected metabolic potentials. Relative expression level was calculated based on the reads per kilobase per million mapped reads (RPKM) of a given selected gene normalized by that of the reference gene *gyrA*. The relative gene expression levels of hydrogenases were selected to indicate the activity of fermentation processes occurred in the two SRMs. Values are presented as means ± SD ($n = 3$). Kruskal-Wallis rank sum test was performed to determine significant differences in the relative gene expression levels between the two strains. * $P < 0.05$. The gene abbreviations are in accordance with those in Fig. 2. RC, respiratory complex. The results are based on three soil samples collected from the

unamended layer of the revegetated tailings. Additional details are presented in Supplementary Table 2−8 and 13.

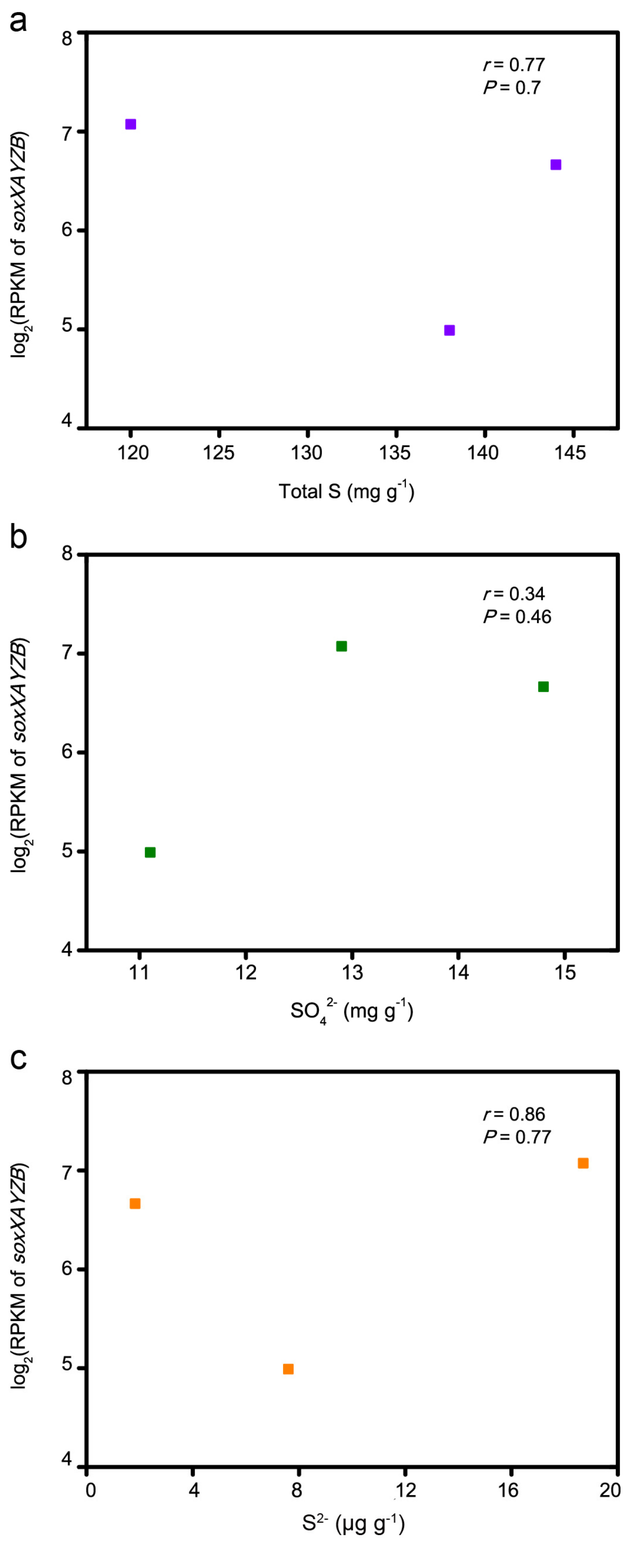

Supplementary figure 11. Correlations between transcript abundance of sox*XAYZB* and concentrations of soil total sulfur (a), sulfate (b) and sulfide (c). Results are based on Pearson correlation analysis.

Supplementary Table 1. Overview of the reductive *dsrAB* -containing MAGs recovered from the mine wasteland.

MAG IDs	Genome bins#	Completeness (%)	Contamination (%)	Genome size (Mb)	Gene no.	Taxonomy (based on GTDB-Tk annotation)	16S rRNA sequence predicted	Relative abundance (%)
Ac.bin1	ULRT4_3.bins.75	98.06	0.86	4.75	3947	Acidobacteriota;Acidobacteriae;Acidobacteriales;Acidobacteriaceae;Terracidiphilus;	Yes	0.055
Ac.bin2	ULRT4_1.bins.77	95.53	1.72	5.11	4196	Acidobacteriota;Acidobacteriae;Acidobacteriales;Acidobacteriaceae;Terracidiphilus;	Yes	0.054
Ac.bin3	ULRT3_2.bins.110	95.47	7.76	6.53	5817	Acidobacteriota;Acidobacteriae;Acidobacteriales;Acidobacteriaceae;Terracidiphilus;	Yes	0.030
De.bin1	ULRT4_3.bins.101	94.64	0.79	3.68	3325	Desulfobacterota_A;Desulfovibrionia;Desulfovibrionales;Desulfovibrionaceae;Desulfovibrio_N;	No	0.051
Ac.bin4	UT3_2.bins.71	94.02	0	4.23	3399	Acidobacteriota;Acidobacteriae;Acidobacteriales;Acidobacteriaceae;Granulicella_A;	No	0.031
Ac.bin5	ULRT4_2.bins.48	92.67	0	5.82	5139	Acidobacteriota;Acidobacteriae;Acidobacteriales;Acidobacteriaceae;Palsa-343;	Yes	0.036
Ac.bin6	UT4_3.bins.137	92.65	0.43	3.73	3093	Acidobacteriota;Acidobacteriae;Acidobacteriales;Acidobacteriaceae;Granulicella_A;	No	0.031
Ac.bin7	ULRT3_3.bins.30	89.78	0.43	4.28	3682	Acidobacteriota;Acidobacteriae;Acidobacteriales;Acidobacteriaceae;Terracidiphilus;	Yes	0.028
Ac.bin8	ULRT3_2.bins.77	88.69	7.76	4.51	4019	Acidobacteriota;Acidobacteriae;Acidobacteriales;Acidobacteriaceae;Terracidiphilus;	No	0.046
Ac.bin9	UT3_3.bins.87	78.4	0	3.15	2801	Acidobacteriota;Acidobacteriae;Acidobacteriales;Acidobacteriaceae;Granulicella_A;	No	0.031
De.bin2	ULRT4_3.bins.66	75.17	0.86	3.02	2975	Desulfobacterota_A;Desulfovibrionia;Desulfovibrionales;Desulfovibrionaceae;Desulfovibrio_F;	Yes	0.024
Ac.bin10	ULRT3_2.bins.124	66.11	0	3.61	3559	Acidobacteriota;Acidobacteriae;Acidobacteriales;Acidobacteriaceae;Terracidiphilus;	Yes	0.020
De.bin3	ULRT4_2.bins.47	65.41	0	2.45	2585	Desulfobacterota_A;Desulfovibrionia;Desulfovibrionales;Desulfovibrionaceae;Desulfovibrio_N;	No	0.036
De.bin4	ULRT4_2.bins.61	63.39	0.81	2.50	2589	Desulfobacterota;Syntrophobacteria;Syntrophobacterales;Syntrophobacteraceae;;	No	0.018
Ac.bin11	ULRT3_2.bins.142	59.77	0	3.15	2649	Acidobacteriota;Acidobacteriae;Acidobacteriales;Acidobacteriaceae;Terracidiphilus;	Yes	0.039
Ac.bin12	ULRT4_2.bins.123	53.76	0	2.58	2618	Acidobacteriota;Acidobacteriae;Acidobacteriales;Acidobacteriaceae;Terracidiphilus;	No	0.046

#The bins were named after the sample from which they were assembled and binned. For example, the name 'ULRT4_3.bins.75' meant that this bin was generated from the metagenomic dataset of the sample ULRT4_3 (representing the third replicate sample collected from the unamended layer of the reclaimed tailings at four years after ecological restoration initiation), as the 75th bin of the genome bins recovered from the mine wasteland.

Supplementary Table 2. Directions of dissimilatory sulfur metabolism for the MAGs recovered in this study.

Putative functions	Genes	MAG No.	MAGs
Sulfate reduction (to sulfide)	*aprBA*, *sat* are present, DsrAB cluster with reductive DsrAB, *dsrD* gene is present, and *dsrEFH* are absent	11	Ac.bin1, Ac.bin2, Ac.bin3, Ac.bin4, Ac.bin5, Ac.bin6, Ac.bin7, Ac.bin11, De.bin1, De.bin2, De.bin3
Sulfite reduction (to sulfide)	DsrAB cluster with reductive DsrAB, *dsrD* gene is present, and *dsrEFH* are absent	2	Ac.bin9, Ac.bin10
Sulfite reduction (to sulfide) incomplete	DsrAB cluster with reductive DsrAB, *dsrD* or *aprAB*, *sat* genes are not entirely present, and *dsrEFH* are absent	3	Ac.bin8, Ac.bin12, De.bin4

Supplementary Table 3. Genes encoding glycoside hydrolases for the sulfate-reducing bacteria and reference species considered in this study.

Glycoside hydrolase family	Ac.bin1	Ac.bin2	Ac.bin3	Ac.bin4	Ac.bin5	Ac.bin6	*Terracidiphilus gabretensis* S55	De.bin1	*Desulfovibrio vulgaris* Hildenborough	*Desulfococcus multivorans* DSM 2059
GH3	9	12	11	10	13	9	10	1	1	1
GH13	11	6	12	6	7	5	7	4	2	3
GH23	4	2	5	2	3	2	3	5	8	4
GH2	4	9	4	5	7	6	6	0	0	0
GH31	5	5	5	5	6	6	3	0	0	0
GH29	6	6	6	4	7	3	3	0	0	0
GH28	4	6	3	6	7	5	3	0	0	0
GH27	3	7	2	7	3	6	4	0	0	0
GH92	2	6	2	5	4	5	3	0	0	0
GH35	5	4	5	3	2	3	3	0	0	0
GH78	2	6	2	5	2	5	0	0	0	0
GH39	3	6	4	3	3	3	5	0	0	0
GH51	4	2	3	2	6	2	2	0	0	0
GH20	3	4	3	3	3	3	3	0	0	0
GH38	1	5	2	3	2	4	2	0	0	0
GH105	2	5	1	2	5	2	2	0	0	0
GH18	3	2	3	2	3	3	4	0	0	0
GH42	3	3	3	3	1	2	1	0	0	0
GH5	1	3	3	2	3	1	3	0	0	0
GH36	2	2	2	1	5	1	4	0	0	0
GH15	2	2	2	2	2	2	2	0	0	0
GH144	2	2	3	2	1	2	1	0	0	0
GH106	0	3	0	4	2	3	1	0	0	0
GH79	1	4	1	2	2	2	1	0	0	0
GH57	1	1	1	2	1	2	1	2	1	0
GH146	1	3	1	2	2	1	1	0	0	0
GH54	1	2	1	2	1	2	2	0	0	0
GH8	2	0	2	1	1	1	2	1	0	0
GH33	0	3	0	2	2	1	0	0	0	0
GH1	1	1	2	1	1	1	1	0	0	0
GH125	1	1	1	1	2	1	1	0	0	0
GH9	3	0	2	0	2	0	1	0	0	0
GH77	1	1	1	1	1	0	1	0	1	0
GH43	1	3	1	0	0	1	5	0	0	0
GH116	0	2	0	3	0	1	3	0	0	0
GH130	1	1	1	1	1	0	1	0	0	0
GH95	1	2	0	1	0	1	2	0	0	0
GH32	1	0	1	1	1	1	2	0	0	0
GH74	2	0	2	0	1	0	0	0	0	0
GH55	1	1	1	0	1	0	2	0	0	0
GH37	1	1	1	0	1	0	0	0	0	0
GH24	0	1	0	1	0	1	1	0	0	1
GH76	0	2	0	1	0	1	2	0	0	0
GH16	0	0	1	2	1	0	0	0	0	0
GH103	0	0	0	0	0	0	0	2	1	1
GH59	0	1	0	1	1	1	0	0	0	0
GH97	1	1	1	0	0	0	1	0	0	0
GH115	0	1	1	0	1	0	1	0	0	0
GH67	1	0	1	0	1	0	0	0	0	0

GH141	1	0	0	1	0	1	0	0	0	0
GH154	0	2	0	0	1	0	0	0	0	0
GH137	0	1	0	1	0	1	0	0	0	0
GH138	0	1	0	1	0	1	0	0	0	0
GH10	0	1	0	0	1	0	1	0	0	0
GH89	0	0	0	1	0	1	1	0	0	0
GH102	0	0	0	0	0	0	0	0	1	1
GH63	1	0	0	0	1	0	0	0	0	0
GH44	1	0	1	0	0	0	1	0	0	0
GH85	0	0	0	1	0	1	0	0	0	0
GH94	0	0	0	0	1	0	1	0	0	0
GH140	0	0	0	0	1	0	0	0	0	0
GH26	1	0	0	0	0	0	0	0	0	0
GH136	0	1	0	0	0	0	0	0	0	0
GH120	0	1	0	0	0	0	0	0	0	0
GH127	0	0	0	0	1	0	0	0	0	0
GH93	0	1	0	0	0	0	0	0	0	0
GH142	0	1	0	0	0	0	1	0	0	0
GH139	0	0	0	0	1	0	0	0	0	0
GH4	0	0	0	0	1	0	0	0	0	0
GH50	0	0	0	0	0	0	0	1	0	0
GH11	1	0	0	0	0	0	0	0	0	0

Supplementary Table 4. Genes encoding hydrogenases for the sulfate-reducing bacteria and reference species considered in this study.

Hydrogenases	Ac.bin1	Ac.bin2	Ac.bin3	Ac.bin4	Ac.bin5	Ac.bin6	*Terracidiphilus gabretensis* S55	De.bin1	*Desulfovibrio vulgaris* Hildenborough	*Desulfococcus multivorans* DSM 2059
FeFe Group A1	0	0	0	0	0	0	0	0	1	0
FeFe Group A2	0	0	0	0	0	0	0	0	1	0
NiFe Group 1a	0	0	0	0	0	0	0	0	2	0
NiFe Group 1b	0	0	0	0	0	0	0	1	1	0
NiFe Group 1d	1	0	1	0	1	0	0	0	0	0
NiFe Group 3d	1	1	1	0	1	0	0	0	0	1
NiFe Group 4c	0	0	0	0	0	0	0	1	1	0
NiFe Group 4e	0	0	0	0	0	0	0	1	1	0

Supplementary Table 5. Genes encoding respiratory complexs for the sulfate-reducing bacteria and reference species considered in this study.

COG	Gene	Function	Ac.bin1	Ac.bin2	Ac.bin3	Ac.bin4	Ac.bin5	Ac.bin6	*Terracidiphilus gabretensis* S55	De.bin1	*Desulfovibrio vulgaris* Hildenborough	*Desulfococcus multivorans* DSM 2059
COG0838	*nuoA*	respiratory complex I (NADH dehydrogenase 1)	2	2	3	2	2	2	2	2	0	1
COG0852	*nuoC*	respiratory complex I (NADH dehydrogenase 1)	2	2	2	2	2	2	2	3	2	1
COG0649	*nuoD*	respiratory complex I (NADH dehydrogenase 1)	2	2	3	2	2	2	2	2	0	0
COG1005	*nuoH*	respiratory complex I (NADH dehydrogenase 1)	2	2	3	2	2	2	2	2	0	1
COG0839	*nuoJ*	respiratory complex I (NADH dehydrogenase 1)	2	2	3	2	2	2	2	2	0	1
COG0713	*nuoK*	respiratory complex I (NADH dehydrogenase 1)	2	2	3	2	2	2	2	2	0	1
COG1009	*nuoL*	respiratory complex I (NADH dehydrogenase 1)	2	2	4	3	2	3	2	2	1	2
COG1008	*nuoM*	respiratory complex I (NADH dehydrogenase 1)	3	3	4	3	3	3	3	3	0	1
COG1007	*nuoN*	respiratory complex I (NADH dehydrogenase 1)	2	2	3	2	2	2	2	2	0	1
COG1905	*nuoE*	respiratory complex I (NADH dehydrogenase 1)	2	3	3	1	2	1	1	1	0	4
COG1894	*nuoF*	respiratory complex I (NADH dehydrogenase 1)	2	3	3	1	3	1	1	1	0	4
COG3383	*nuoG*	respiratory complex I (NADH dehydrogenase 1)	4	4	6	5	5	5	1	3	0	7
COG0377	*nuoB*	respiratory complex I (NADH dehydrogenase 1)	2	2	3	2	1	2	2	1	0	0
COG1143	*nuoI*	respiratory complex I (NADH dehydrogenase 1)	3	4	4	3	4	3	2	5	6	1
COG1034	*nuoG_a*	respiratory complex I (NADH dehydrogenase 1)	0	0	1	0	0	0	0	1	0	1
COG1252	*ndh*	respiratory complex I (NADH dehydrogenase 2)	1	1	1	0	1	0	1	1	2	1
COG0029	*sdhA/frdA*	respiratory complex II (succinate dehydrogenase)	1	1	1	1	1	1	1	1	1	1
COG0479	*sdhB/frdB*	respiratory complex II (succinate dehydrogenase)	1	1	3	1	1	1	1	2	2	1
COG2009	*sdhC*	respiratory complex II (succinate dehydrogenase)	1	1	1	0	0	0	1	0	0	0
COG1053	*sdhA*	respiratory complex II (succinate dehydrogenase)	2	3	2	1	2	1	1	3	4	2
COG0723	*petC*	respiratory complex III (quinol-cytochrome-c reductase)	6	5	7	2	5	2	1	0	0	1
COG1290	*petB/petD*	respiratory complex III (quinol-cytochrome-c reductase)	5	4	6	2	6	2	1	0	0	0
COG0243	*actB1*	respiratory complex III (quinol-cytochrome-c reductase)	2	2	3	2	3	2	1	6	9	5
COG0437	*actB*	respiratory complex III (quinol-cytochrome-c reductase)	4	3	12	3	4	4	0	3	10	7
COG5557	*actC*	respiratory complex III (quinol-cytochrome-c reductase)	2	2	2	2	2	2	1	2	3	5
COG1622	*coxM*	respiratory complex IV (low-affinity terminal oxidase)	0	0	0	0	2	0	1	1	0	0
COG0843	*coxN*	respiratory complex IV (low-affinity terminal oxidase)	3	2	4	2	3	1	2	1	1	0
COG1845	*coxO*	respiratory complex IV (low-affinity terminal oxidase)	4	3	4	3	3	3	3	1	1	0
COG3125	*ctaF*	respiratory complex IV (low-affinity terminal oxidase)	0	0	0	0	0	0	0	1	0	0
COG1271	*cydA*	respiratory complex IV (bd-type high-affinity terminal oxidase)	2	2	3	2	3	2	0	2	3	4
COG1294	*cydB*	respiratory complex IV (bd-type high-affinity terminal oxidase)	1	1	2	1	2	1	0	1	1	2
COG3278	*ccoN*	respiratory complex IV (cbb3-type high-affinity terminal oxidase)	0	1	1	1	0	1	0	0	0	1
COG2993	*ccoO*	respiratory complex IV (cbb3-type high-affinity terminal oxidase)	0	0	1	0	0	0	0	0	0	0
COG0056	*atpA*	respiratory complex V (F-type ATP synthase)	1	2	1	1	1	1	1	1	1	2
COG0055	*atpD*	respiratory complex V (F-type ATP synthase)	1	2	1	1	1	1	1	1	1	2
COG0712	*atpH*	respiratory complex V (F-type ATP synthase)	1	2	1	1	1	1	1	1	1	1
COG0355	*atpC*	respiratory complex V (F-type ATP synthase)	1	2	1	1	1	1	1	1	1	2
COG0224	*atpG*	respiratory complex V (F-type ATP synthase)	1	2	1	1	1	1	1	1	2	2
COG0356	*atpB*	respiratory complex V (F-type ATP synthase)	1	2	1	1	1	1	1	1	1	3
COG0711	*atpF*	respiratory complex V (F-type ATP synthase)	1	2	1	2	2	2	2	2	2	5

Supplementary Table 6. Core genes responsible for chemotaxis and motility for the sulfate-reducing bacteria and reference species considered in this study.

KO	Gene	Function	Ac.bin1	Ac.bin2	Ac.bin3	Ac.bin4	Ac.bin5	Ac.bin6	*Terracidiphilus gabretensis* S55	De.bin1	*Desulfovibrio vulgaris* Hildenborough	*Desulfococcus multivorans* DSM 2059
PF00015	*MCP*	methyl-accepting chemotaxis protein	2	1	0	1	6	1	1	34	28	0
K03412	*cheB*	protein-glutamate methylesterase/glutaminase	2	1	1	1	5	1	1	5	2	0
K00575	*cheR*	chemotaxis protein methyltransferase CheR	1	1	0	1	5	1	1	4	2	0
K13924	*cheBR*	vo-component system, chemotaxis family, CheB/CheR fusion protei	1	0	2	0	2	0	0	2	1	1
K03407	*cheA*	two-component system, chemotaxis family, sensor kinase CheA	2	1	0	1	4	1	1	4	3	0
K03408	*cheW*	purine-binding chemotaxis protein CheW	1	1	0	1	4	1	1	4	4	0
K03413	*cheY*	wo-component system, chemotaxis family, chemotaxis protein CheY	2	1	1	3	6	3	2	9	5	0
K02387	*flgB*	flagellar basal-body rod protein FlgB	1	1	1	0	2	1	1	1	1	0
K02388	*flgC*	flagellar basal-body rod protein FlgC	1	1	1	0	2	0	1	1	2	0
K02389	*flgD*	flagellar basal-body rod modification protein FlgD	1	1	1	0	1	0	1	2	1	0
K02390	*flgE*	flagellar hook protein FlgE	1	1	1	0	2	1	1	2	2	0
K02391	*flgF*	flagellar basal-body rod protein FlgF	0	0	0	0	1	0	0	0	0	0
K02392	*flgG*	flagellar basal-body rod protein FlgG	1	1	1	0	2	0	1	2	2	0
K02396	*flgK*	flagellar hook-associated protein 1 FlgK	1	1	1	0	2	0	1	2	1	0
K02397	*flgL*	flagellar hook-associated protein 3 FlgL	1	1	1	0	2	0	1	2	1	0
K02400	*flhA*	flagellar biosynthesis protein FlhA	1	1	1	0	3	0	1	1	1	0
K02401	*flhB*	flagellar biosynthetic protein FlhB	1	1	1	0	2	0	1	1	1	0
K02406	*fliC*	flagellin	2	1	2	0	2	0	1	3	4	0
K02407	*fliD*	flagellar hook-associated protein 2	1	1	1	0	2	0	1	2	1	0
K02408	*fliE*	flagellar hook-basal body complex protein FliE	1	1	1	0	2	0	1	1	1	0
K02409	*fliF*	flagellar M-ring protein FliF	1	1	1	0	2	0	1	1	1	0
K02410	*fliG*	flagellar motor switch protein FliG	1	1	1	0	2	0	1	1	1	0
K02411	*fliH*	flagellar assembly protein FliH	1	2	1	0	2	0	1	1	1	0
K02412	*fliI*	flagellum-specific ATP synthase	1	1	1	0	2	0	1	1	1	0
K02416	*fliM*	flagellar motor switch protein FliM	1	1	1	0	2	0	1	1	1	0
K02417	*fliN*	flagellar motor switch protein FliN	2	1	1	0	3	0	1	1	1	0
K02419	*fliP*	flagellar biosynthetic protein FliP	1	1	1	0	2	0	1	1	1	0
K02420	*fliQ*	flagellar biosynthetic protein FliQ	1	1	1	0	2	0	1	1	1	0
K02421	*fliR*	flagellar biosynthetic protein FliR	1	1	1	0	2	0	1	1	1	0
K02556	*motA*	chemotaxis protein MotA	2	2	2	2	4	1	2	5	4	0
K02557	*motB*	chemotaxis protein MotB	2	2	2	1	3	0	2	5	5	0
K02650	*pilA*	type IV pilus assembly protein PilA	0	0	0	1	0	1	0	0	0	1
K02652	*pilB*	type IV pilus assembly protein PilB	1	1	1	1	1	1	1	1	1	0
K02653	*pilC*	type IV pilus assembly protein PilC	1	1	1	1	1	1	1	1	1	1
K02654	*pilD, pppA*	leader peptidase (prepilin peptidase) / N-methyltransferase	1	1	1	2	1	2	1	1	2	1
K02662	*pilM*	type IV pilus assembly protein PilM	1	1	1	1	1	1	1	0	0	1
K02663	*pilN*	type IV pilus assembly protein PilN	1	1	1	1	0	1	1	0	0	0
K02651	*flp, pilA*	pilus assembly protein Flp/PilA	1	1	1	0	5	0	0	0	2	1
K02669	*pilT*	twitching motility protein PilT	0	0	0	1	1	1	0	3	1	2
K02664	pilO	type IV pilus assembly protein PilO	0	0	0	0	0	0	0	0	0	1
K02665	pilP	type IV pilus assembly protein PilP	0	0	0	0	0	0	0	0	0	1
K02666	pilQ	type IV pilus assembly protein PilQ	0	0	0	0	0	0	0	1	1	1

Supplementary Table 7. Classification* of methyl-accepting chemotaxis proteins (MCP) for the sulfate-reducing bacteria and reference species considered in this study.

	Ia		Ib	II	IIIm	IIIc	IVa	IVb
	Cluster I	Cluster II						
Ac.bin1	0	0	0	0	0	0	1	1
Ac.bin2	1	0	0	0	0	0	0	0
Ac.bin3	0	0	0	0	0	0	0	0
Ac.bin4	0	0	0	0	0	0	1	0
Ac.bin5	2	0	0	0	0	0	1	3
Ac.bin6	0	0	0	0	0	0	1	0
Terracidiphilus gabretensis S55	1	0	0	0	0	0	0	0
De.bin1	18	9	0	0	2	2	0	3
Desulfovibrio vulgaris Hildenborough	17	10	0	0	1	0	0	0
Desulfococcus multivorans DSM 2059	0	0	0	0	0	0	0	0

*Results are based on membrane topology and ligand-binding domain.

Supplementary Table 8. Genes encoding oxidative stress for the sulfate-reducing bacteria and reference species considered in this study.

KO	Key proteins	Function	Ac.bin1	Ac.bin2	Ac.bin3	Ac.bin4	Ac.bin5	Ac.bin6	*Terracidiphilus gabretensis* S55	De.bin1	*Desulfovibrio vulgaris* Hildenborough	*Desulfococcus multivorans* DSM 2059
K03781	catalase (EC 1.11.1.6)	hydrogen peroxide:hydrogen peroxide oxidoreductase	0	0	0	0	2	0	0	1	1	1
K00425	cytochrome bd oxygen reductase (EC 7.1.1.7)	Cytochrome bd oxygen reductase (cydA)	1	1	2	1	3	1	0	1	1	2
K00426		Cytochrome bd oxygen reductase (cydB)	1	1	2	1	2	1	0	1	1	2
K02274	cytochrome c oxidase (ferrocytochrome c:oxygen oxidoreductase, EC 7.1.1.9)	coxA, ctaD; cytochrome c oxidase subunit I	2	2	3	2	3	1	2	0	1	0
K02275		coxB, ctaC; cytochrome c oxidase subunit II	3	2	3	2	6	1	3	0	1	0
K02276		coxC, ctaE; cytochrome c oxidase subunit III	3	2	3	2	3	2	3	0	1	0
K01738	cysteine synthase (CysK; EC 2.5.1.47)	cysK; cysteine synthase	2	1	2	2	3	1	1	1	1	1
K00533	[Fe] hydrogenase (EC 1.12.7.2)	ferredoxin hydrogenase large subunit	0	0	0	0	0	0	0	0	2	0
K00534		ferredoxin hydrogenase small subunit	0	0	0	0	0	0	0	0	1	0
K00428	cytochrome c peroxidase (Ccp; EC 1.11.1.5)	cytochrome c peroxidase	0	1	0	0	1	0	0	0	0	0
COG1592;PF02915	NADH peroxidases (EC 1.11.1.1)	Rubrerythrin1;Nigerythrin	3	1	3	0	2	0	1	5	5	7
COG0491;PF00753		Rubrerythrin2	2	2	2	2	5	2	5	2	2	3
K00432	glutathione peroxidase (EC 1.11.1.9)	gpx, btuE, bsaA; glutathione peroxidase	2	1	2	1	0	1	4	0	0	0
K11065	thioredoxin peroxidase (Tpx; EC 1.11.1.15)	tpx; thioredoxin-dependent peroxiredoxin	0	0	0	0	0	0	0	1	1	1
K03564	thioredoxin-dependent peroxiredoxin (EC 1.11.1.24)	BCP	1	2	2	2	2	2	2	1	1	1
PF00753;PF00258;COG0426	rubredoxin-oxygen oxireductase	rubredoxin-oxygen oxireductase (ROO)	1	0	0	0	0	0	0	2	1	4
K05919	superoxide reductase (EC 1.15.1.2)	dfx; superoxide reductase	0	0	0	0	0	0	0	1	1	1
K16627	superoxide dismutase (EC 1.15.1.1)	Copper/zinc superoxide dismutase	0	0	0	0	0	0	0	0	0	0
K04565		Copper/zinc superoxide dismutase	0	0	0	1	0	1	1	0	0	0
K04564		Iron/manganese superoxide dismutase	1	1	1	1	1	1	1	1	1	2
K00518		Nickel-containing superoxide dismutase	0	0	0	0	0	0	0	0	0	1

Supplementary Table 9. Virus-host linkages information for the sulfate-reducing bacteria and reference species considered in this study.

Viral sequence ID[#]	VirSorter_category	vContact2_cluster	No. of sequences in the cluster	Taxonomy	Virion-associated gene (i.e., capsid, terminase or portal) detected in viral sequences	Phage transposase & integrase detected in viral sequences	Phage tail component in viral sequences	Host genome bin	Host taxonomy (based on GTDB-Tk annotation)
Ac.bin1.1	Category_5	VC_335_0	5	unassigned	+	-	-	Ac.bin1	Acidobacteriota;Acidobacteriae;Acidobacteriales;Acidobacteriaceae;Terracidiphilus;
	Category_5	singleton	-	unassigned	+	+	-		
Ac.bin2.1	Category_5	VC_335_0	5	unassigned	+	-	-	Ac.bin2	Acidobacteriota;Acidobacteriae;Acidobacteriales;Acidobacteriaceae;Terracidiphilus;
Ac.bin3.1	Category_5	VC_335_0	5	unassigned	+	-	-	Ac.bin3	Acidobacteriota;Acidobacteriae;Acidobacteriales;Acidobacteriaceae;Terracidiphilus;
Ac.bin5.1	Category_5	VC_335_0	5	unassigned	+	-	-	Ac.bin5	Acidobacteriota;Acidobacteriae;Acidobacteriales;Acidobacteriaceae;;
Ac.bin11.1	Category_5	singleton	-	unassigned	+	+	-	Ac.bin11	Acidobacteriota;Acidobacteriae;Acidobacteriales;Acidobacteriaceae;Terracidiphilus;
T. gabretensis.1	Category_5	VC_335_0	5	unassigned	+	-	-	*Terracidiphilus gabretensis* S55	Acidobacteriota;Acidobacteriae;Acidobacteriales;Acidobacteriaceae;Terracidiphilus;
D. vulgaris.1	Category_4	singleton	-	Caudovirales	+	+	+	*Desulfovibrio vulgaris* Hildenborough	Desulfobacterota; Desulfovibrionia; Desulfovibrionales; Desulfovibrionaceae; Nitratidesulfovibrio; Nitratidesulfovibrio vulgaris
D. vulgaris.2	Category_4	singleton	-	Caudovirales	+	+	+		
D. vulgaris.3	Category_5	VC_61_0	9	Caudovirales; Myoviridae	+	-	+		
D. vulgaris.4	Category_5	VC_336_0	3	Caudovirales	+	+	+		
D. vulgaris.5	Category_5	singleton	-	Caudovirales	-	+	+		
D. vulgaris.6	Category_5	VC_336_0	3	Caudovirales	-	+	+		
D. vulgaris.7	Category_5	VC_336_0	3	Caudovirales	+	+	+		
D. multivorans.1	Category_5	singleton	-	unassigned	-	+	-	*Desulfococcus multivorans* DSM 2059	Desulfobacterota; Desulfobacteria; Desulfobacterales; Desulfococcaceae; Desulfococcus; Desulfococcus multivorans
D. multivorans.2	Category_5	singleton	-	Caudovirales	-	-	+		
D. multivorans.3	Category_5	singleton	-	unassigned	-	+	-		

[#]Viral sequences were named according to their host. For example, Ac.bin1.1 represents the first viral sequence of Ac.bin1.

Supplementary Table 10. Glycoside hydrolases (GHs) encoded by viruese infecting the sulfate-reducing bacteria and reference species considered in this study.

Viral sequence ID	Gene	Host	CAZy family	Signalp	Phyre2 Annotation	Protein Data Bank Best Hit ID	Model Confidence %	Sequence % Identity	Query Coverage (% of protein aligned)	Protein Phyre ID	Protein Model Organism
Ac.bin3.1	NODE_263_length_121426_cov_5.268051_26	Ac.bin3	GH105	N	D-4,5-unsaturated β-glucuronyl hydrolase (EC: 3.2.1.172)	4CE7	100	42	72	6219b7372294dff3	*Nonlabens ulvanivorans*
D. vulgaris.2	NZ_CABHLV010000001.1_980	*Desulfovibrio vulgaris* Hildenborough	GH23	Y (1-32)	chitinase (EC 3.2.1.14)	3W6D	100	18	78	cab4a76fdc34f6f0	*Ralstonia sp.* A-471
D. vulgaris.5	NZ_CABHLV010000001.1_1306	*Desulfovibrio vulgaris* Hildenborough	GH23	Y (1-31)	chitinase (EC 3.2.1.14)	3W6D	100	16	79	99acf6a7696aa132	*Ralstonia sp.* A-471

Supplementary Table 11. Viral methyl-accepting chemotaxis proteins (MCPs) and superoxide dismutase (SOD) encoded by viruese infecting the sulfate-reducing bacteria and reference species considered in this study.

Virus	Gene	Host	size (aa)	MCP_Category	Interpro Annotation	Function	start_aa	end_aa	Phyre2 Annotation	Protein Data Bank Best Hit ID	Model Confidence %	Sequence % Identity	Query Coverage (% of protein aligned)	Protein Phyre ID	Protein Model Organism
D. vulgaris.1	NZ_CABHLV010000001.1_2702		813	Ia (Cluster II)	PF17201	Cache 3/Cache 2 fusion domain	34	318	Cache domain of methyl-accepting chemotaxis protein	3C8C	99.7	17	34	97c8efb277d8aa81	*Vibrio cholerae*
					PF00672	HAMP domain	341	390							
					PF08448	PAS fold	405	513							
					PF00015	Methyl-accepting chemotaxis protein (MCP) signalling domain	590	773							
D. vulgaris.1	NZ_CABHLV010000001.1_2799	*Desulfovibrio vulgaris* Hildenborough	626	Ia (Cluster I)	PF08269	Cache domain 2	54	227	Cache-like sensor domain mediating C2 (glycolate and acetate) and C3 (propionate and pyruvate) carboxylates sensing	5G4Y	99.5	43	23	74ba713b5a5e3de9	*Pseudomonas syringae*
					PF00672	HAMP domain	251	301							
					PF00015	Methyl-accepting chemotaxis protein (MCP) signalling domain	409	591							
D. vulgaris.2	NZ_CABHLV010000001.1_943		804	Ia (Cluster II)	PF02743	Double Cache domain 1	49	284	Double CACHE (dCACHE) domain mediating lactate sensing	5WBF	99.9	34	11	6cee65165d56e32c	*Helicobacter pylori* 26695
					PF00672	HAMP domain	338	384							
					PF13426	PAS domain	405	507							
					PF00015	Methyl-accepting chemotaxis protein (MCP) signalling domain	587	768							
D. multivorans.2	CP015381.1_2500	*Desulfococcus multivorans* DSM 2059	157	/	PF09055	Nickel-containing superoxide dismutase	27	150	Nickel-containing superoxide dismutase, NiSOD	1T6U	100	16	75	ee61aa0bf878be49	*Streptomyces coelicolor*

Supplementary Table 12. Selected physico-chemical properties of soil samples used for metatranscriptomic analysis of this study.

	ULRT1	ULRT2	ULRT3
pH	7.65	7.80	7.75
Eh (mV)	190	191	194
Total C (mg g^{-1})	5.18	1.90	2.71
Total S (mg g^{-1})	144	120	138
SO_4^{2-} (mg g^{-1})	14.8	12.9	11.1
S^{2-} (μg g^{-1})	1.83	18.7	7.60
Fe^{2+} (μg g^{-1})	10.6	9.19	5.22

Supplementary Table 13. Genes responsible for denitrification and fermentation illustraed in Supplementary Figure 10 in this study.

KO	Function	Ac.bin5	De.bin1
K00370	nitrate reductase 1	0	0
K00371	nitrate reductase 1	0	0
K00374	nitrate reductase 1	0	0
K02567	nitrate reductase (cytochrome)	0	0
K02568	nitrate reductase (cytochrome)	0	0
K00368	nitrate reductase (NO-forming)	0	0
K15864	nitrate reductase (NO-forming)	0	0
K04561	nitric oxide reductase	2	1
K02305	nitric oxide reductase	0	0
K00376	nitrous-oxide reductase	0	0

Supplementary Table 14. The information of metatranscriptomic datasets used in this study.

	ULRT1	ULRT2	ULRT3
No. of metatranscripomic raw reads	42179613	89679037	47502326
No. of metatranscripomic quality-trimmed reads	40357389	86004585	43953294
No. of quality-trimmed cDNA reads	32042524	48888842	34021435
No. of quality-trimmed cDNA reads that could be mapped to the 16 MAGs recovered in this study	66601	136109	214175

Supplementary References

1. Müller, A. L., Kjeldsen, K. U., Rattei, T., Pester, M. & Loy, A. Phylogenetic and environmental diversity of DsrAB-type dissimilatory (bi)sulfite reductases. *ISME J.* **9**, 1152–1165 (2015).
2. Anantharaman, K. et al. Expanded diversity of microbial groups that shape the dissimilatory sulfur cycle. *ISME J.* **12**, 1715–1728 (2018).
3. Mizuno, N., Voordouw, G., Miki, K., Sarai, A. & Higuchi, Y. Crystal structure of dissimilatory sulfite reductase D (DsrD) protein–possible interaction with B- and Z-DNA by its winged-helix motif. *Structure.* **11**, 1133–1140 (2003).
4. Fu, R., Wall, J. D. & Voordouw G. DcrA, a c-Type Heme-containing methyl-accepting protein from *Desulfovibrio vulgaris* Hildenborough, senses the oxygen concentration or redox potential of the environment. *J. Bacteriol.* **176**, 344–350 (1994).
5. Ray, J. et al. Exploring the role of CheA3 in *Desulfovibrio vulgaris* Hildenborough motility. *Front Microbiol.* **5**, 77 (2014).
