# Supplementary material for: Metagenomic and metatranscriptomic insights into sulfate-reducing bacteria in a revegetated acidic mine wasteland
Source: NPJ Biofilms Microbiomes. 2022 Sep 6;8:71. doi: 10.1038/s41522-022-00333-9 (PMC9448743; doi:10.1038/s41522-022-00333-9)
Supplement: Supplementary file 2 — Reporting Summary [file 41522_2022_333_MOESM2_ESM.pdf]

nature portfolio

Corresponding author(s): Jie-Liang Liang
Last updated by author(s): Aug 1, 2022

Reporting Summary

Nature Portfolio wishes to improve the reproducibility of the work that we publish. This form provides structure for consistency and transparency in reporting. For further information on Nature Portfolio policies, see our Editorial Policies and the Editorial Policy Checklist.

Statistics

For all statistical analyses, confirm that the following items are present in the figure legend, table legend, main text, or Methods section.

n/a	Confirmed	
☐	☒	The exact sample size (*n*) for each experimental group/condition, given as a discrete number and unit of measurement
☐	☒	A statement on whether measurements were taken from distinct samples or whether the same sample was measured repeatedly
☐	☒	The statistical test(s) used AND whether they are one- or two-sided<br>*Only common tests should be described solely by name; describe more complex techniques in the Methods section.*
☐	☒	A description of all covariates tested
☐	☒	A description of any assumptions or corrections, such as tests of normality and adjustment for multiple comparisons
☐	☒	A full description of the statistical parameters including central tendency (e.g. means) or other basic estimates (e.g. regression coefficient) AND variation (e.g. standard deviation) or associated estimates of uncertainty (e.g. confidence intervals)
☐	☒	For null hypothesis testing, the test statistic (e.g. *F*, *t*, *r*) with confidence intervals, effect sizes, degrees of freedom and *P* value noted<br>*Give P values as exact values whenever suitable.*
☐	☒	For Bayesian analysis, information on the choice of priors and Markov chain Monte Carlo settings
☐	☒	For hierarchical and complex designs, identification of the appropriate level for tests and full reporting of outcomes
☐	☒	Estimates of effect sizes (e.g. Cohen's *d*, Pearson's *r*), indicating how they were calculated

Our web collection on statistics for biologists contains articles on many of the points above.

Software and code

Policy information about availability of computer code

Data collection

The genome sequences of D. vulgaris Hildenborough, Desulfococcus multivorans DSM 2059 and Terracidiphilus gabretensis S55 were downloaded from the NCBI Genbank database. Other data were generated in this study.

Data analysis

Open source tools:
1.Assembling of short reads: SPAdes (version 3.9.0)
2.ORFs predicting: Prodigal (version 2.6.3)
3.Construction of genomes from metagenome: MetaBAT (version 0.30.3)
4.Improvement of metagenome-assembled genomes (MAGs): RefineM (version 0.0.14)
5.Check quality of MAGs: CheckM (version 1.0.4)
6.Functional annotation: Diamond (version 0.9.24.125), HMMER (version 3.2) and dbCAN2 meta server (https://bcb.unl.edu/dbCAN2/)
7.Sequence alignment and filtering: MUSCLE (version 3.8.1551) TrimAL (version 1.2rev59)
8.Construction of phylogenetic trees: RAxML (version 8.2.12) and PhyloPhlAn (version 0.99)
9.Reads mapping to genes sequences and MAGs: BBmap (version 38.44)
10.Editing of phylogenetic tree topology: the Interactive Tree of Life online interface (https://itol.embl.de/)
11.Taxonomic assignment of MAGs: GTDB-Tk (version 1.0.2)
12.Phage analysis: VirSorter (version 1.0.6) and vConTACT2 (version 0.9.17)
13.Protein modeling: PHYRE2 (http://www.sbg.bio.ic.ac.uk/phyre2/html/page.cgi?id=index)
14.Metatranscriptomic reads filtering: fastp (version 0.21.0) and SortMeRNA (version 4.2.0)
15. Statistical analysis: R software 3.6.3

For manuscripts utilizing custom algorithms or software that are central to the research but not yet described in published literature, software must be made available to editors and reviewers. We strongly encourage code deposition in a community repository (e.g. GitHub). See the Nature Portfolio guidelines for submitting code & software for further information.

Data

Policy information about availability of data

All manuscripts must include a data availability statement. This statement should provide the following information, where applicable:

- Accession codes, unique identifiers, or web links for publicly available datasets
- A description of any restrictions on data availability
- For clinical datasets or third party data, please ensure that the statement adheres to our policy

The metagenomic and metatranscriptomic data analysed in this study were deposited at EMBL under accession numbers PRJEB31441 and PRJEB42658, and the MAGs reported in this study have been deposited in GenBank under accession numbers SAMN15699825 and SAMN15808056–70. The datasets generated during and/or analyzed during the current study are available from the corresponding author on reasonable request.

Human research participants

Policy information about studies involving human research participants and Sex and Gender in Research.

Reporting on sex and gender	Not relevant to our study
Population characteristics	Not relevant to our study
Recruitment	Not relevant to our study
Ethics oversight	Not relevant to our study

Note that full information on the approval of the study protocol must also be provided in the manuscript.

Field-specific reporting

Please select the one below that is the best fit for your research. If you are not sure, read the appropriate sections before making your selection.

☐ Life sciences ☐ Behavioural & social sciences ☒ Ecological, evolutionary & environmental sciences

For a reference copy of the document with all sections, see nature.com/documents/nr-reporting-summary-flat.pdf

Ecological, evolutionary & environmental sciences study design

All studies must disclose on these points even when the disclosure is negative.

Study description	In this study, we used genome-resolved metagenomics and metatranscriptomics to explore the diversity and survival strategies of SRMs in a suboxic mine soil. The results not only improve our understanding of the diversity of microorganisms involved in dissimilatory sulfate reduction in terrestrial environments under constantly oxic/hypoxic conditions, but also reveal the metabolic potential of SRM-infecting viruses as participants in the terrestrial S biogeochemical cycle.
Research sample	We selected a revegetated acidic mine wasteland as our study site. This site consisted of three different habitats: an amended layer of revegetated tailings (0-10 cm, ALRT), an unamended layer of revegetated tailings (11-20 cm, ULRT) and unrevegetated tailings (UT). In total, we collected 18 soil samples, as we considered three soil types (i.e., ALRT, ULRT, and UT), two sampling dates (i.e., in July 2016 and 2017) and three replicates for each soil type on each sampling date.
Sampling strategy	On each sampling date, three reclaimed plots were randomly selected for sampling. In each plot, one soil sample was collected from ALRT (0-10 cm) and from ULRT (11-20 cm). One soil sample was also collected from UT next to each of the three sampled plots at a depth of 0–10 cm as controls. Soil samples were collected using a stainless steel trowel.
Data collection	Soil DNA was extracted using PowerSoil DNA isolation kit (Mobio Laboratories Inc., USA) with modification. The DNA quality was determined with a NanoDrop 2000 spectrophotometer (Thermo Scientific, USA). Each DNA sample was purified and then used to construct a shotgun library (~500 bp average insert size), which was sequenced (150 or 250 bp paired-end reads) using an Illumina MiSeq sequencer (Illumina, USA).<br><br>Total cellular RNA was extracted using the RNeasy PowerSoil Total RNA kit (QIAGEN, USA) according to the manufacturer's instructions. Total RNA was transported to the Magigene Company (Guangzhou, China) on dry ice for subsequent rRNA subtraction, cDNA synthesis, library construction, and sequencing with an Illumina NovaSeq platform (paired-end 150-bp mode).
Timing and spatial scale	Soil samples were collected in July 2016 and 2017, at local scale.
Data exclusions	No data was excluded.

Reproducibility	Three replicates for each soil type were collected.
Randomization	On each sampling date, three reclaimed plots were randomly selected for sampling.
Blinding	During data analysis, blinding was conducted by only taking the sample ID into account within the same sample group.

Did the study involve field work? ☒ Yes ☐ No

Field work, collection and transport

Field conditions	The study site is characterized by a subtropical humid monsoon climate, with a mean annual precipitation of 1426 mm and a mean annual temperature of 17.0°C. The soils from three habitats were rich in sulfate (1.80-25.9 g SO42- kg-1 dry soil) and were under constantly oxic/hypoxic conditions (as indicated by a soil Eh range of approximately 180-680 mV).
Location	29°40′52″N, 115°49′21″E
Access & import/export	Import/export is not relevant to our study.
Disturbance	No disturbance.

Reporting for specific materials, systems and methods

We require information from authors about some types of materials, experimental systems and methods used in many studies. Here, indicate whether each material, system or method listed is relevant to your study. If you are not sure if a list item applies to your research, read the appropriate section before selecting a response.

Materials & experimental systems

n/a	Involved in the study	
☒	☐	Antibodies
☒	☐	Eukaryotic cell lines
☒	☐	Palaeontology and archaeology
☒	☐	Animals and other organisms
☒	☐	Clinical data
☒	☐	Dual use research of concern

Methods

n/a	Involved in the study	
☒	☐	ChIP-seq
☒	☐	Flow cytometry
☒	☐	MRI-based neuroimaging
